# New gene signature from the dominant infiltration immune cell type in osteosarcoma predicts overall survival

**DOI:** 10.1038/s41598-023-45566-6

**Published:** 2023-10-25

**Authors:** Liping Gong, Xifeng Sun, Ming Jia

**Affiliations:** 1 Department of Academic Research, The Secondary Hospital, Cheeloo College of Medicine, Shandong University, Jinan, 250033 China; 2 Department of Clinical Laboratory, The Second Hospital, Cheeloo College of Medicine, Shandong University, Jinan, 250033 China; 3 Department of Cancer Center, The Secondary Hospital, Cheeloo College of Medicine, Shandong University, Jinan, 250033 China

**Keywords:** Bone cancer, Computational biology and bioinformatics

## Abstract

The immune microenvironment of osteosarcoma (OS) has been reported to play an important role in disease progression and prognosis. However, owing to tumor heterogeneity, it is not ideal to predict OS prognosis by examining only infiltrating immune cells. This work aimed to build a prognostic gene signature based on similarities in the immune microenvironments of OS patients. Public datasets were used to examine the correlated genes, and the most consistent dominant infiltrating immune cell type was identified. The LASSO Cox regression model was used to establish a multiple-gene risk prediction signature. A nine-gene prognostic signature was generated from the correlated genes for M0 macrophages and then proven to be effective and reliable in validation cohorts. Signature comparison indicated the priority of the signature. Multivariate Cox regression models indicated that the signature risk score is an independent prognostic factor for OS patients regardless of the Huvos grade in all datasets. In addition, the results of the association between the signature risk score and chemotherapy sensitivity also showed that there was no significant difference in the sensitivity of any drugs between the low- and high-risk groups. A GSEA of GO and KEGG pathways found that antigen processing- and presentation-related biological functions and olfactory transduction receptor signaling pathways have important roles in signature functioning. Our findings showed that M0 macrophages were the dominant infiltrating immune cell type in OS and that the new gene signature is a promising prognostic model for OS patients.

## Introduction

Osteosarcoma (OS) is the most common primary malignant tumor in children and adolescents. However, its incidence rate is quite low, with approximately 3610 new cases diagnosed annually worldwide^1,2^. Although the mortality rate of OS has dramatically decreased due to neoadjuvant multiagent systemic chemotherapy, advanced surgical techniques, and precision radiotherapy, the survival rate is still not satisfactory, especially for those patients with tumor metastasis and recurrence^3,4^. It has been challenging to improve the prognosis of OS.

Numerous scientists have studied the gene mechanisms and treatments of OS. The development of humanized in vitro and humanized mouse models with similar tumor microenvironments has greatly facilitated related research^5,6^. In vitro 3D tumor models showed that MAPK, TGFβ/SMAD, PI3K/AKT, JAK/STAT, Notch and Hedgehog signaling transition molecules exhibit significantly increased expression^7^. Nigris et al. also reported that OS cells cultured in scaffolds showed a dramatic increase in angiogenic factors such as PDGFB, TGFB1, VEGFA and VEGFB^8^. HER2, CXCR4 and HIF2α were found to be typical biomarkers involved in tumor growth and the homing of cancer cells to distant sites^9^. Humanized in vitro models of osteosarcoma also showed improved drug resistance to doxorubicin compared with scaffold-free spheroids^10^. Regarding treatment, Dimas et al. found that targeting the farnesylation of Ras by self-assembling nanoparticles encapsulating zoledronic acid simultaneously inhibited the Ras/ERK1/2/HIF-1α and Ras/Akt/mTOR axes, leading to reduced growth and increased intratumour necro-apoptosis and T-cell infiltration of chemo-immune-resistant osteosarcomas^11^. Targeting mitochondrial complex I prevented HIF1-MIF activation, leading to TAM accumulation and vascular architecture remodeling and ultimately resulting in the inhibition of osteosarcomas grown in the humanized model^12^. Immunotherapy drugs such as nivolumab have also been investigated in a humanized mouse model by Zheng et al., and the results showed that the nivolumab-treated group exhibited a similar primary tumor volume and growth rate but a significantly lower lung metastasis rate than the control group. Further analysis showed that CD4 + and CD8 + lymphocytes were more frequently observed in the lungs of the nivolumab-treated group than in the lungs of the control group, but no statistically significant differences were observed in the primary tumors from both groups^13^. All these results indicated that immunotherapy targeting the tumor microenvironment may be a promising strategy for osteosarcoma treatment.

The immune microenvironment of OS has been investigated since the accidental discovery of tumor responses following bacterial toxin treatment. Its functions are diverse and complex and have not been fully understood until now^14,15^. Previous results showed that osteosarcomas are infiltrated mainly by macrophages and T cells^16–18^. Studies have shown that high infiltration levels of macrophages and CD8 + T cells are associated with reduced metastasis and improved survival in OS^19–21^. In contrast, high infiltration levels of antigen-presenting cells have been reported to lead to unfavorable outcomes^22^. In recent years, several studies have focused on the detailed molecular mechanisms; for example, Wang et al. found that OS tumor cells could release PD-L1 to promote the metastatic process by inhibiting the immune response^23^. Troyer et al. reported that indoleamine dioxygenase could inhibit dendritic cells from producing neoantigens, thus indirectly leading to immune escape^24^. A previous study also reported that all-trans retinoic acid could inhibit M2 polarization of macrophages to repress the OS lung metastatic process^25^. In addition, the VEGF, IL-10A, TGF-β, and STAT3 pathways have also been found to facilitate the immunosuppressive microenvironment by influencing bone marrow-derived suppressor cells, macrophages and stromal fibroblasts^15^. Together, these data highlight the important role of the immune microenvironment in patients with OS. However, the mechanism by which infiltrating immune cells regulate the pathogenesis and development of OS remains largely unknown. In addition, it is not ideal to predict the prognosis and therapeutic response solely using infiltrating immune cells of OS patients. With the appearance of high-dimensional datasets and advanced bioinformatics algorithms^26–28^, it is now possible to analyze the comprehensive interactions between biological phenotypes and the tumor immune microenvironment, thus facilitating research on the molecular characteristics affecting immune cell infiltration, the response to immunotherapy, and the prognosis of OS patients.

In our study, we gained a deeper understanding of the OS immune microenvironment by utilizing OS cohorts from four public databases, which showed that M0 macrophages were the most consistently infiltrating immune cell type. We then screened for genes correlated with M0 macrophages in the Therapeutically Applicable Research to Generate Effective Treatments (TARGET) and GSE21257 datasets under the supposition that dominant immune cells have more vital effects on OS prognosis. A prognostic immune signature was built by the least absolute shrinkage and selection operator (LASSO) Cox model using the abovementioned M0 macrophage-correlated genes from the TARGET training cohort. The predictive values of this model were further validated in two other independent testing cohorts and verified by comparing them to previous prognostic models. Then, we attempted to identify the related signaling pathways, intrinsic molecular subtypes, hot immune-targeted gene expression, and distributions of immune cells between each risk score subtype. Furthermore, the relationship between the risk score and predicted chemotherapy sensitivity was also assessed.

## Methods

### Data collection

Gene chip expression data and the clinical information of OS patients from the TARGET database were obtained from the website https://ocg.cancer.gov/programs/target/data-matrix on May 1, 2022. Osteosarcoma was used as the only key word to select suitable datasets published up to May 2022 for our study on the Gene Expression Omnibus (GEO) datasets website (https://www.ncbi.nlm.nih.gov/gds/). Then, the datasets were searched by the following inclusion criteria: (1) the diagnoses were pathologically confirmed; (2) the dataset had Homo sapiens samples; (3) the platform contained whole-genome information; (4) the dataset included patient survival data; and (5) the sample size of the datasets was more than 30. After that, only the GSE21257, GSE16091, and GSE39055 datasets were included in our study. Therefore, the normalized mRNA expression data and survival data of the above datasets were downloaded from GEO (https://www.ncbi.nlm.nih.gov/geo/query/acc.cgi?acc=GSE21257, https://www.ncbi.nlm.nih.gov/geo/query/acc.cgi?acc=GSE16091, https://www.ncbi.nlm.nih.gov/geo/query/acc.cgi?acc=GSE39055).

If the same patient provided two or more tumor samples to those datasets, only the data corresponding to the primary lesion were selected according to the sample numbers. All patients were included in our study. Gene symbols were extracted from the provided documents from those dataset websites. We conducted signal intensity normalization across the arrays of the above datasets using the normalizeBetweenArrays function from the limma package in R software.

### Tumor immune cell infiltration calculation

The infiltration levels of 22 immune cells in the TARGET, GSE21257, GSE16091, and GSE39055 datasets were quantified by the “CIBERSORT” R package using the 1,000 permutations method. The reference immune cell signatures were downloaded from the Supplementary Information on the article’s website (https://www.nature.com/articles/nmeth.3337#MOESM207)^29–31^. The ESTIMATE score, stromal score, and immune score of each patient were assessed by the “ESTIMATE” R package using the gene expression data from each patient sample^32^. All parameters in the R equation throughout the calculation process were set to default.

### M0 macrophage correlated gene screening

This section attempts to select genes associated with M0 macrophages. Pearson's correlation analysis was used to search for M0 macrophage-correlated genes with cutoffs of |correlation coefficient|> 0.3 and *P* value < 0.05 in the TARGET and GSE21257 datasets, which have a relatively large sample size. Next, we merged those correlated genes from the two datasets to obtain a full correlated gene list.

### Gene signature construction and validation

Gene signatures were generated by inputting M0 macrophage-correlated genes into the LASSO Cox regression model using the TARGET dataset. The “glmnet” package in R was used to complete the regression process. After the genes were selected, a multivariate Cox analysis was used to calculate the corresponding coefficients. The signature risk score was calculated by the sum of the products of each gene and its corresponding coefficients as follows: score = (CPE*0.7021) + (FHL5*− 0.9794) + (GBP1*− 0.4718) + (GNLY*0.9393) + (GPR82*− 1.8384) + (IL18RAP*− 1.9630) + (LILRA2*0.4310) + (NDRG4*− 0.7661) + (PLB1*− 0.6822). Moreover, the risk scores for the GSE21257 and GSE39055 datasets were also calculated using the same method with the coefficients derived from the TCGA dataset for validation. OS patients from each dataset were divided into two groups (low- and high-risk groups) according to the median value of the risk score to maximize the statistical power and provide the most reliable results based on a fixed sample size in those databases^33^. An overall survival analysis between the two groups was conducted using Kaplan–Meier curves with Wilcoxon log-rank tests. Multivariate Cox regression models were applied to test the independent predictive value of our signature. The prognostic accuracy of the risk score in different datasets was determined using the time-dependent receiver operating characteristic curve (ROC) and incident/dynamic (I/D) area under the curve (AUC) through the “timeROC” and “risksetROC” packages in R separately^34^.

### Gene signature comparison

We screened the studies using the keyword “gene signature prognosis osteosarcoma” on PubMed. In addition, the inclusion criteria were as follows: (1) the journal impact factor was more than 5 and had a good reputation; (2) the online publication date range was from March 01, 2020, to March 01, 2022; and (3) the gene signature was constructed by messenger RNA to be easily validated in other datasets. After the studies were selected, the gene signatures were recalculated by a multivariate Cox proportional hazards model using data in a training cohort. Kaplan–Meier curves, time-dependent ROCs and I/D AUCs of the gene signatures using validation cohorts were chosen to compare the prognostic accuracy.

### Gene set enrichment analysis (GSEA)

GSEA was launched to study the biological functions of our gene signature in the TARGET, GSE21257, and GSE39055 datasets using the “clusterProfiler” R package based on the GO and KEGG analyses individually. The GO functions and KEGG pathways with P values less than 0.05 in each dataset were merged. Finally, the biological functions with consistent positive or negative values of enrichmentScore were reserved to find the real difference of GO functions and KEGG pathways between low- and high-risk groups discriminated by our signatures.

### Correlation between signature risk score and chemotherapy sensitivity

The IC50 values of chemotherapy drugs (bleomycin, cisplatin, doxorubicin, etoposide, and methotrexate) in each osteosarcoma sample in the TARGET, GSE21257, and GSE39055 datasets were accessed through the “pRRophetic” package in R^35^, which was built based on the Genomics of Drug Sensitivity in Cancer database (www.cancerRxgene.org)^36^. Then, the samples were classified into low- or high-risk groups by the median of our gene signature risk score. Finally, the IC50 values of chemotherapy drugs between different groups were analyzed by the Mann‒Whitney rank test. A *P* value < 0.05 was defined as statistically significant.

### Statistical analysis

We conducted all statistical analyses with R version 4.0.5 (R Foundation for Statistical Computing, Vienna, Austria) and GraphPad Prism 6.01 (GraphPad Software, Inc., San Diego, CA, USA). OS patients were divided into two groups by the median of our signature risk score in the TARGET, GSE21257, and GSE39055 datasets. Violin plots were performed in Hiplot (https://hiplot.com.cn), a comprehensive web platform for scientific data visualization. The comparison of 22 immune cells, immunotherapy-targeted genes, ESTIMATE scores, stromal scores, and immune scores between low- and high-risk groups were analyzed using a Mann‒Whitney rank test. Unless otherwise specified, a *P* value < 0.05 was defined as statistically significant.

### Ethics approval

Our study is based on open-source data (TRGET and GEO). Ethical review and approval were not required for the study on human participants in accordance with the local legislation and institutional requirements. 

### Consent to participate

All methods were carried out in accordance with the relevant guidelines and regulations.

## Results

### Clinical characteristics of the studied datasets

**Figure 1:**
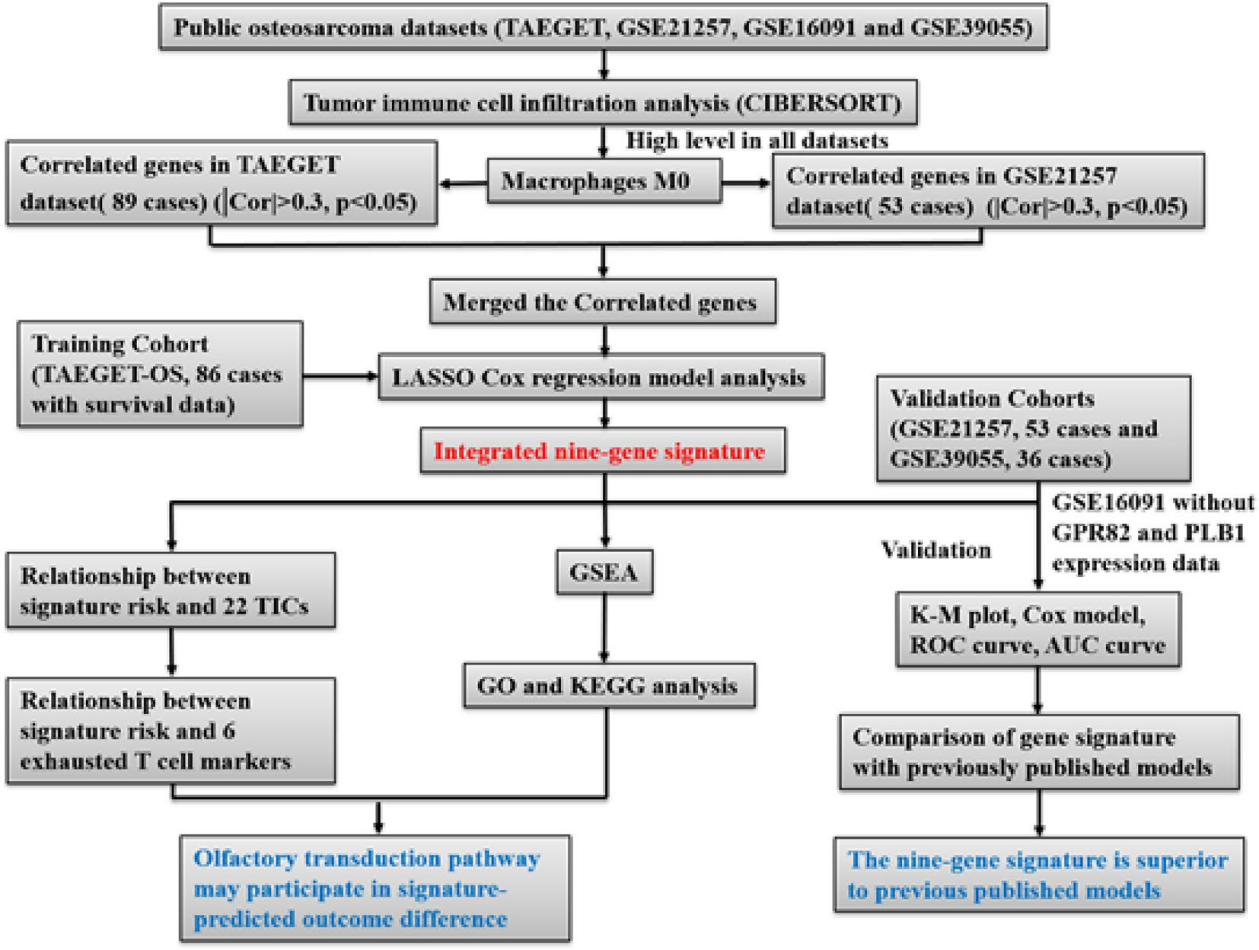
A schematic diagram of dataset analysis.

Figure 1 shows the flowchart of this study. The clinical characteristics of OS patients in the TARGET, GSE21257, GSE16091, and GSE39055 datasets are shown in Table 1. In the TARGET and GSE21257 datasets, the age, sex, tumor location, and metastatic status at diagnosis were similar. The GSE21257 dataset contained the most detailed clinical information except for radiotherapy treatment and had a similar Huvos grade distribution as the GSE39055 dataset. GSEGSE16091 only included survival information. The median overall time and survival curves (Supplemental Figure 1) were close to each other in all datasets.

**Table 1:** Characteristics of OS patients in the TARGET, GSE21257, GSE16091 and GSE39055 datasets.

Variables	TARGET		GSE21257		GSE16091		GSE39055	
	Num	Percent (%)	Num	Percent (%)	Num	Percent (%)	Num	Percent (%)
All	89	100.0	53	100.0	34	100.0	37	100.0
Age								
≤ 15	48	53.9	21	39.6			29	78.4
> 15	41	46.1	32	60.4			8	21.6
Unknown	0	0.0	0	0.0	34	100.0	0	0.0
Sex								
Male	51	57.3	34	64.2			20	54.1
Female	38	42.7	19	35.8			17	45.9
Unknown	0	0.0	0	0.0	34	100.0	0	0.0
Tumor location								
Femur	41	46.1	27	50.9				
Fibula	8	9.0	2	3.8				
Humerus	4	4.5	8	15.1				
Tibia	22	24.7	15	28.3				
Others	14	15.7	0	0.0				
Unknown	0	0.0	1	1.9	34	100.0	37	100.0
Histological subtype								
Chondroblastic			6	11.3				
Fibroblastic			5	9.4				
Osteoblastic			32	60.4				
Others			10	18.9				
Unknown	89	100.0	0	0.0	34	100.0	37	100.0
Status at diagnosis								
Nonmetastatic	66	74.2	39	73.6				
Metastatic	23	25.8	14	26.4				
Unknown	0	0.0	0	0.0	34	100.0	37	100.0
Huvos grade								
I–II	19	21.3	29	54.7			24	64.9
III–IV	17	19.1	18	34.0			13	35.1
Unknown	53	59.6	6	11.3	34	100.0	0	0.0
Definitive surgery								
Yes	46	51.7						
No	0	0.0						
Unknown	43	48.3	53	100.0	34	100.0	37	100.0
Status								
Death	30	33.7	23	43.4	15	44.1	10	27.0
Censored	57	64.0	30	56.6	19	55.9	27	73.0
Unknown	2	2.2	0	0.0	0	0.0	0	0.0 59
Survival time (months)								
Known	86	96.6	53	100.0	34	100.0	36	97.3
Unknown	3	3.4	0	0.0	0	0.0	1	2.7
Median	128.7		189		125.1		151.0	
Follow-up time (months)								
Median	61.5		91		96.8		59	
Range	2.5–194.7		4.0–246.0		0.8–161.2		2.7–200.9	

### Estimation of tumor immune cell infiltration in osteosarcoma

**Figure 2:**
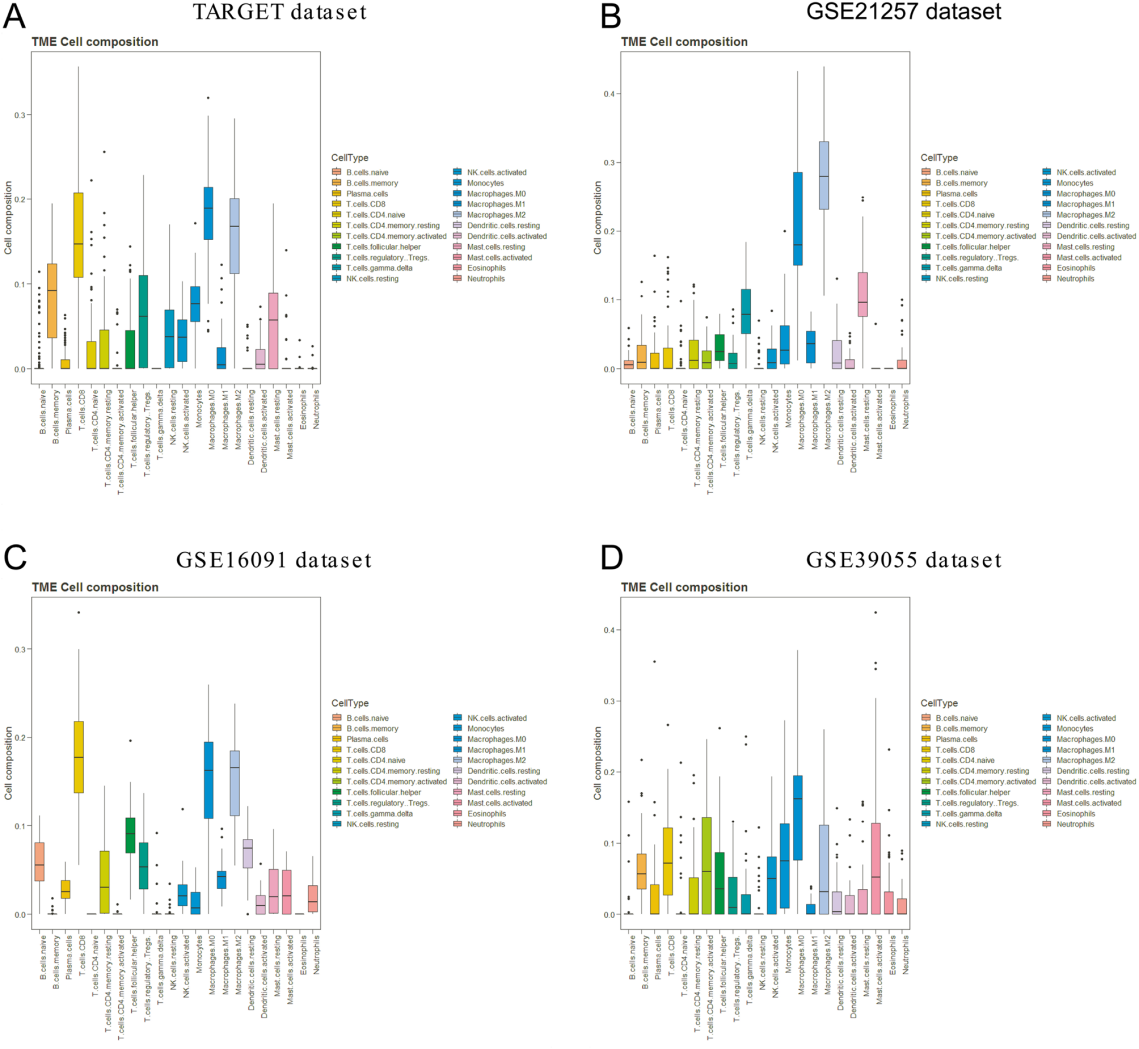
Estimation of tumor immune cell infiltration in osteosarcoma by the CIBERSORT algorithm. The percentage of infiltration of 22 immune cell types in the TARGET (**A**), GSE21257 (**B**), GSE16091 (**C**), and GSE39055 (**D**) datasets showed that M0 macrophages were the dominant immune cell type.

To obtain the landscape of tumor immune cell infiltration, we applied the CIBERSORT algorithm to the selected datasets. As shown in Fig. 2, the major immune cells in the TARGET and GSE16091 datasets were CD8 + T cells and M0 and M2 macrophages, while the major immune cells in the GSE21257 dataset were M0 and M2 macrophages. For the GSE39055 dataset, M0 macrophages were the most dominant infiltrating immune cells. Other immune cell infiltration levels were quite low in all datasets. In general, M0 macrophages were the dominant immune cells across all datasets. As shown in Supplemental Figure 1, the survival difference between each dataset was not significant, and we believe that M0 macrophages are influential on the overall survival of osteosarcoma. Therefore, the M0 macrophage-associated genes were screened by the methods described in the “**Methods and Materials”,** and the selected genes are shown in Supplemental Table 1.

#### Construction and validation of prognostic gene signature for osteosarcoma

**Figure 3:**
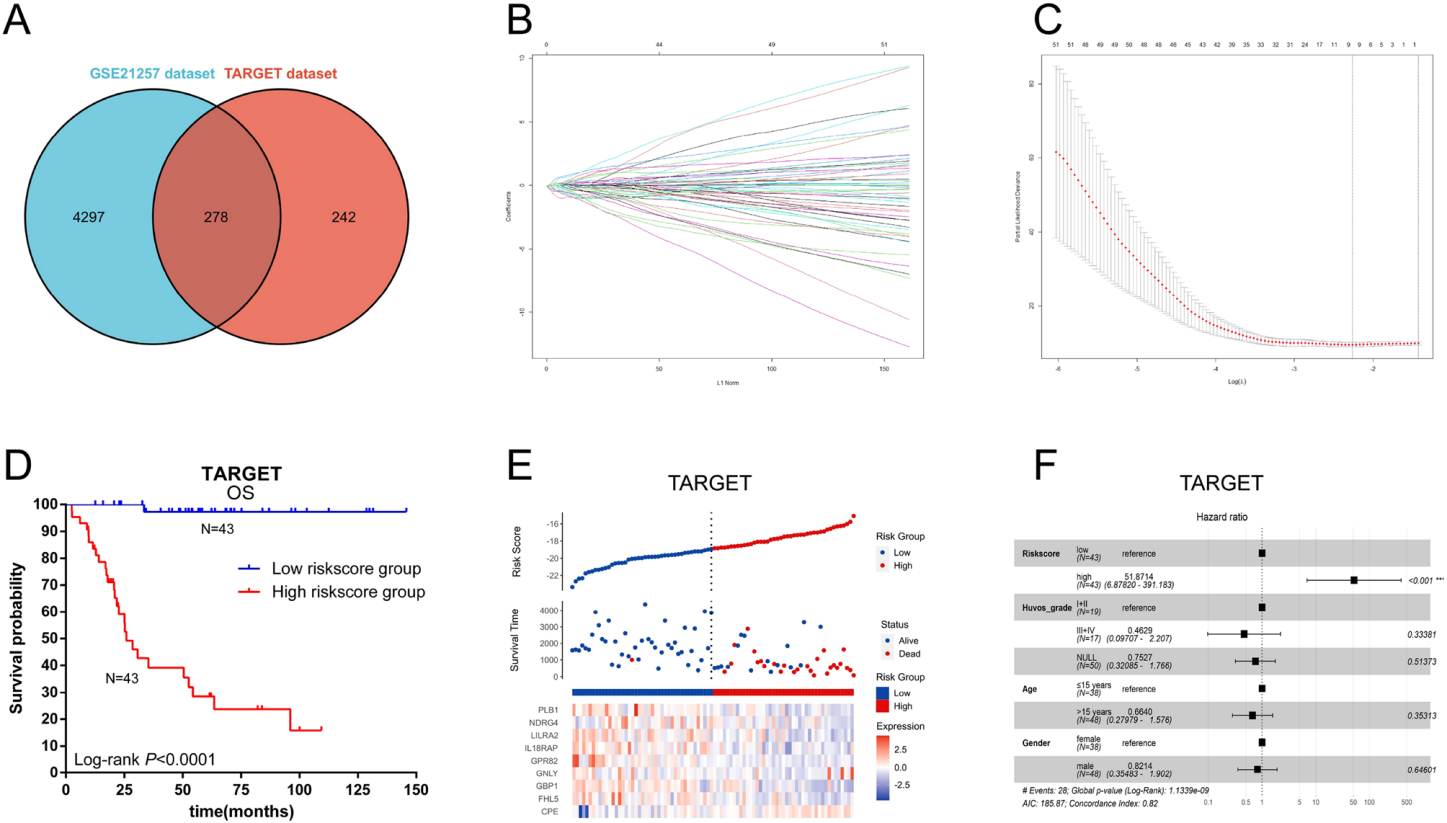
Construction of a prognostic gene signature. (**A**) Venn diagram of the M0 macrophage-associated genes in the TARGET and GSE21257 datasets. The numbers in each area represent the gene numbers in each group. (**B**) Cross-validation for tuning parameter screening upon LASSO regression analysis. (**C**) Screening of the optimal parameter (lambda) at which the vertical lines were drawn. (**D**) Kaplan‒Meier overall survival analysis of the gene signature risk score in OS of the TARGET dataset. (**E**) Distribution of signature gene expression profiles along with survival status in different signature risk score groups in TARGET datasets. (**F**) Forest plot showing the results of multiple factors in the Cox regression analysis of the gene signature risk score with other clinical characteristics in OS of the TARGET dataset.

**Figure 4:**
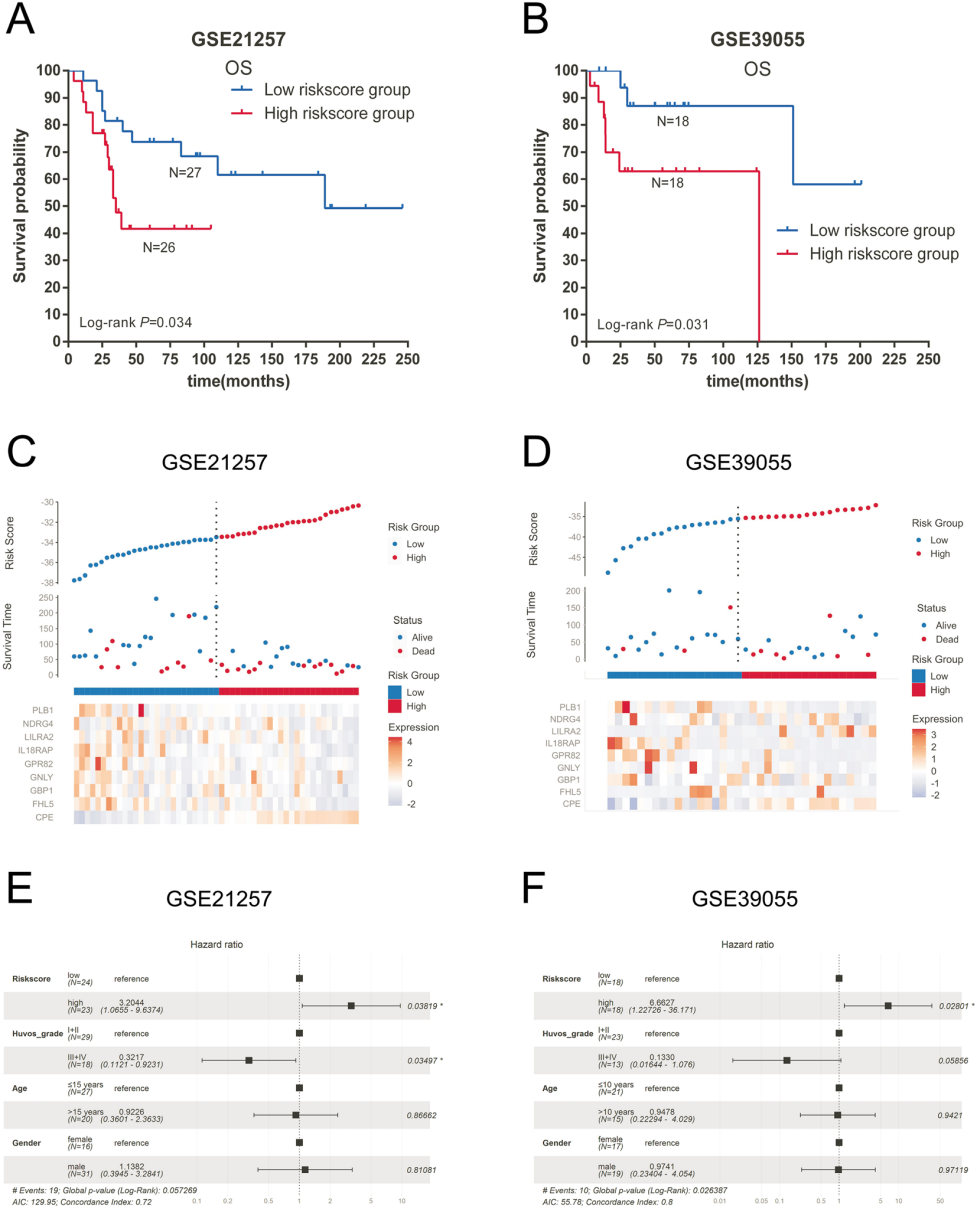
Validation of the nine-gene prognostic signature. Kaplan‒Meier overall survival analysis of the gene signature risk score in OS from the GSE21257 (**A**) and GSE39055 (**B**) datasets. Distribution of signature gene expression profiles along with survival status in different signature risk score groups in the GSE21257 (**C**) and GSE39055 (**D**) datasets. Forest plot shows the results of multiple factors in the Cox regression analysis of the gene signature risk score with other clinical characteristics in overall survival from the GSE21257 (**E**) and GSE39055 (**F**) datasets.

**Figure 5:**
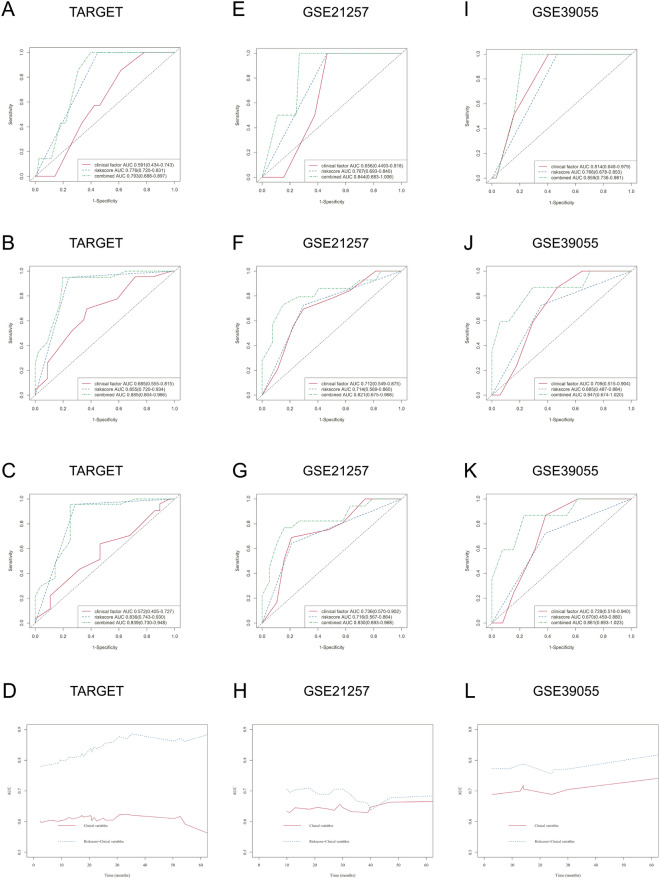
Prognostic values of the signature risk score in the training and validation cohorts. Time-dependent ROCs at 1 (**A**), 3 (**B**), and 5 (**C**) years and I/D AUC (**D**) for OS in the TARGET dataset. Time-dependent ROCs at 1 (**E**), 3 (**F**), and 5 (**G**) years and I/D AUC (H) for OS in the GSE21257 dataset. Time-dependent ROCs at 1 (**I**), 3 (**J**), and 5 (**K**) years and I/D AUC (L) for OS in the GSE39055 dataset. ROC, receiver operating characteristic curve; I/D AUC, incident/dynamic area under the curve.

The above selected genes were used to build a prognostic signature in OS patients through LASSO Cox regression analysis by the TARGET dataset with 86 patients as the discovery cohort (Fig. 3A). An optimal 9-gene prognostic signature was made (Fig. 3B,C)). The biological functions and the coefficients of signature genes are shown in Table 2. The signature risk scores are equal to the sum of the product of the expression value and coefficient of each gene. We chose only the GSE21257 and GSE39055 datasets as the validation cohort to measure the prognostic value of the signature-based risk score, and the GSE16091 dataset was excluded owing to the absence of gene expression data for GPR82 and PLB1. We used the log-rank test to study the association of the risk score with survival in the TARGET dataset. As expected, high-risk patients had significantly shorter survival than low-risk patients (log-rank test, *P* < 0.0001) (Fig. 3D). The same tendency was confirmed in the GSE21257 and GSE39055 datasets (log-rank test, *P* = 0.034 and* P* = 0.031) (Fig. 4A,B). In addition, the AUCs of the time-dependent ROCs including the signature risk score and selected clinical factors (age, sex, and Huvos grade) reached 0.793, 0.885, and 0.839 for 1-, 3-, and 5-year OS in the TARGET dataset, respectively, which were higher than the AUCs of the time-dependent ROCs including only clinical factors (Fig. 5A, B, C). Similar results were also observed in the GSE21257 and GSE39055 datasets (Fig. 5E,F,G,I,J,K).

**Table 2:** Prognostic genes obtained from the LASSO Cox regression model.

Gene symbol	Description	Risk coefficient
CPE	Carboxypeptidase E	0.7021
FHL5	Four and a half LIM domains 5	− 0.9794
GBP1	Guanylate binding protein 1	− 0.4718
GNLY	Granulysin	0.9393
GPR82	G protein-coupled receptor 82	− 1.8384
IL18RAP	Interleukin 18 receptor accessory protein	− 1.963
LILRA2	Leukocyte immunoglobulin like receptor A2	0.431
NDRG4	NDRG family member 4	− 0.7661
PLB1	Phospholipase B1	− 0.6822

We compared the C index of I/D AUC to assess the discriminative accuracy of the prediction model with and without the signature risk score. The results showed that the C index increased to 0.831 (95% CI, 0.750–0.868 *P* < 0.0001), 0.690 (95% CI, 0.527–0.754; *P* < 0.0001), and 0.775 (95% CI, 0.584–0.840; *P* < 0.0001) when the signature risk score was added to the prognostic model in the TARGET, GSE21257, and GSE39055 datasets (Fig. 5D,H,L). Figure 3E and Fig. 4C,D show the distribution of signature gene expression profiles and survival statuses in different risk score groups for OS in all datasets. Figure 3F and Fig. 4E,F show that the high-risk score was significantly associated with a risk of increased mortality in OS patients in the multivariate Cox regression models after adjusting for age, sex, and Huvos grade in all datasets (HR = 51.871, 3.204 and 6.663, 95% CI = 6.898–391.183, 1.066–9.637 and 1.227–36.171, *P* < 0.001, = 0.038 and = 0.028, respectively), which indicates that the signature risk score is an independent prognostic factor for OS patients.

### Investigation into the differences in immune cells, immunotherapy-targeted genes, and immune scores between high- and low-risk groups

**Figure 6:**
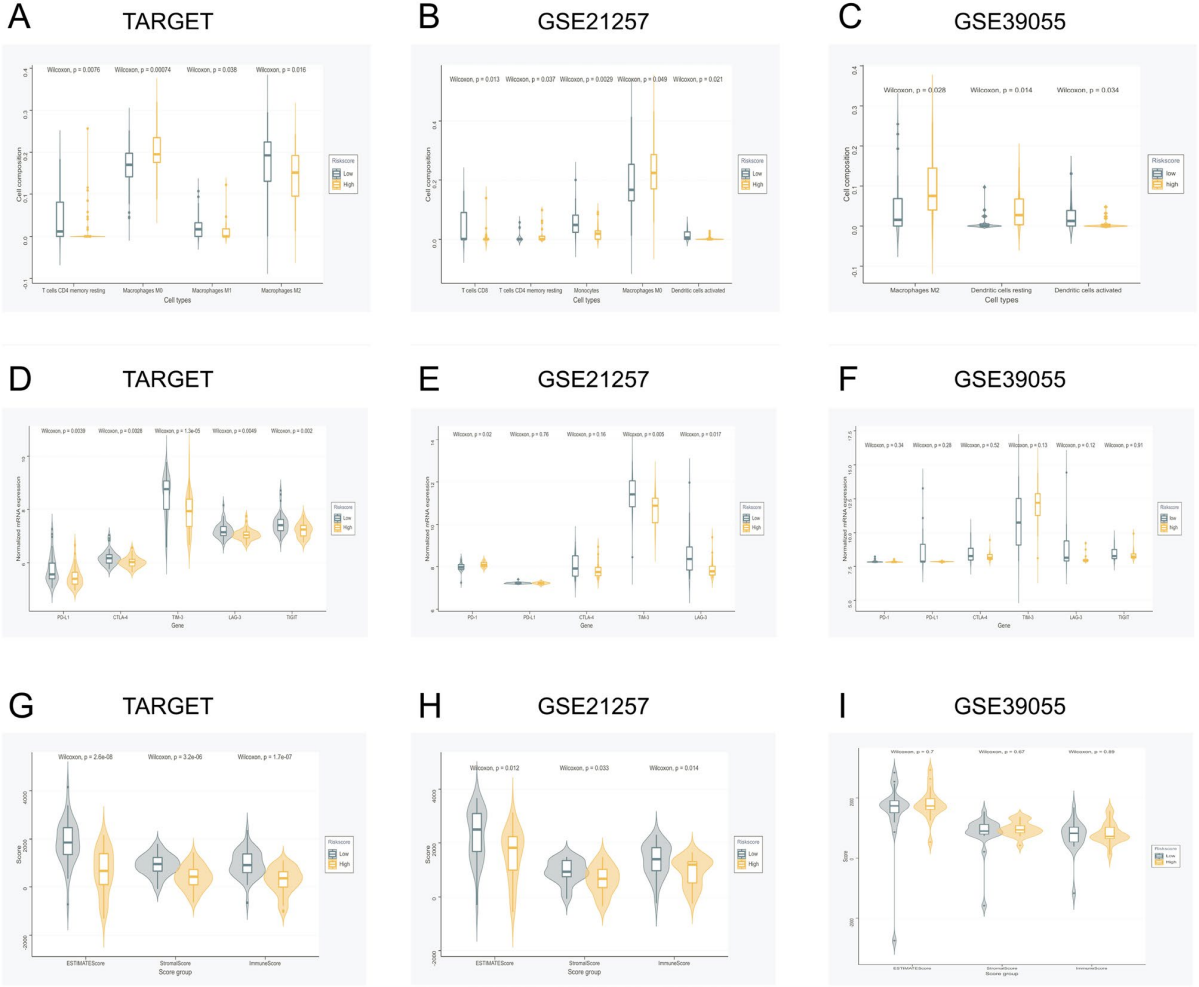
Association between immune cell infiltration levels, immunotherapy-targeted gene expression, ESTIMATE immune scores, and signature risk score of OS. The immune cell infiltration types found to be significantly associated with the signature risk score of OS in the TARGET (**A**), GSE21257 (**B**), and GSE39055 (**C**) datasets. Association between immunotherapy-targeted gene expression and the signature risk score of OS in the TARGET (**D**), GSE21257 (**E**), and GSE39055 (**F**) datasets. Association between ESTIMATE immune scores and signature risk scores of OS in the TARGET (**G**), GSE21257 (**H**), and GSE39055 (**I**) datasets.

As the signature risk score reflects the tumor immune activity of OS, we further investigated the differences in immune cells, immunotherapy-targeted genes, and immune scores between different risk groups from all datasets. The immune cell infiltration results were inconsistent between the three datasets (Supplemental Figure 2). M0 macrophages were found to be higher in high-risk groups than in low-risk groups in the TARGET and GSE21257 datasets (Fig. 6A, B). Activated dendritic cells were found to be lower in the high-risk groups than in the low-risk groups in the GSE21257 and GSE35099 datasets (Fig. 6B, C). For the results of six hot immunotherapy-targeted genes and immune scores, no significant differences were found in the GSE35099 datasets (**6F, I**), which may be due to its relatively small sample size. TIM-3, LAG-3 and all immune scores were all found to be lower in the high-risk groups than in the low-risk groups in the TARGET and GSE21257 datasets (Fig. 6D,E,G,H). In summary, our signature risk score may reflect the immune activity of osteosarcoma. In addition, LAG3 may be the most effective immunotherapy target because its expression was relatively high in all datasets (Fig. 6D,E,F). Owing to the low incidence rate of OS, the OS datasets did not contain large sample sizes. Therefore, adjustments for multiple testing were not made to reserve important clues.

### Comparison of gene signatures

**Figure 7:**
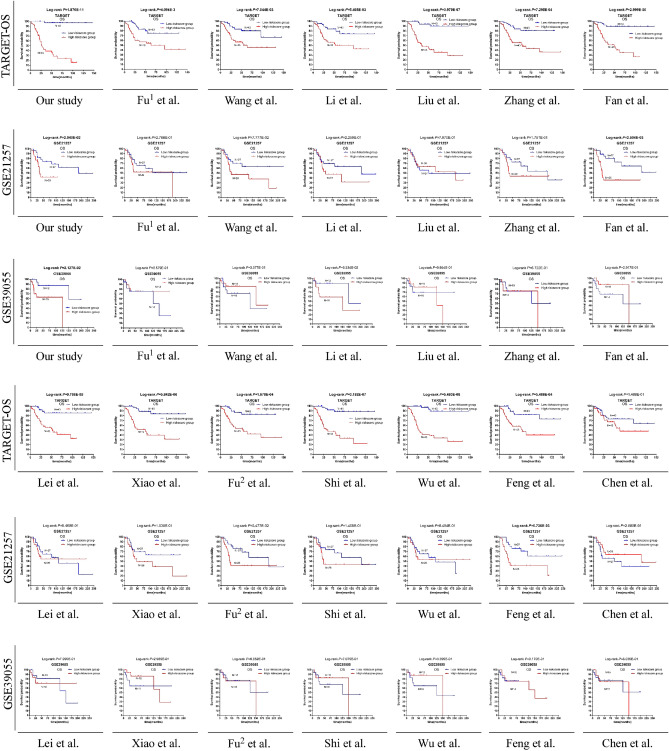
Comparisons of the gene signature with previously published gene signatures in the TARGET, GSE21257, and GSE39055 datasets using the Kaplan–Meier estimator. The results are in bold and considered significant if the *P* value < 0.05.

**Figure 8:**
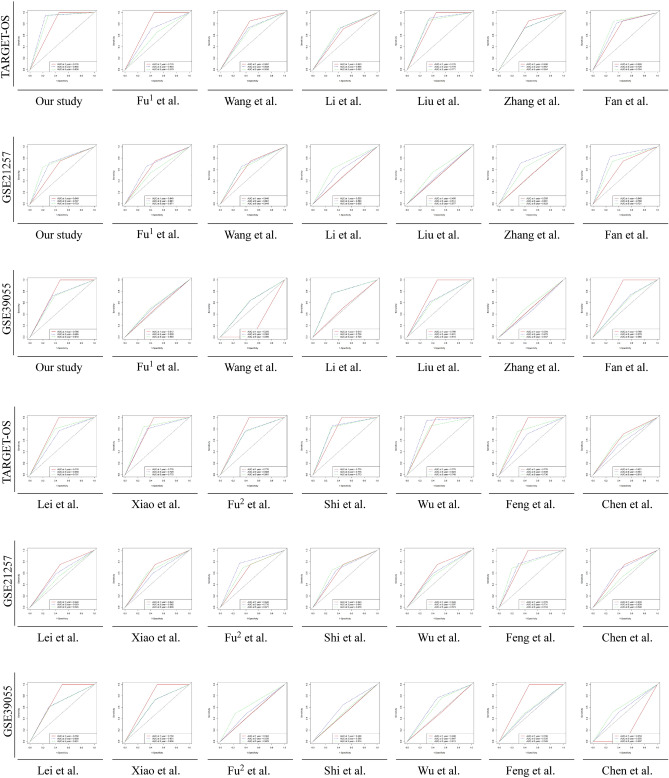
Comparisons of the gene signature with previously published gene signatures in the TARGET, GSE21257, and GSE39055 datasets using time-dependent ROC curves.

Thirteen previously reported studies were included in the comparison after the selection process, which are shown in Table 3. Kaplan–Meier curves (Fig. 7) and univariate Cox models (Table 4) showed that only the difference in overall survival for the nine-gene signature model was significant in the training and testing cohorts. Additionally, the AUCs of the time-dependent ROCs of the signature risk score were also found to be greater than the AUCs of the time-dependent ROCs of previous signatures in the TARGET and GSE39055 datasets (Fig. 8). The results showed that our nine-gene prognostic signature is more robust than previously reported gene signatures.

**Table 3:** Candidate research for comparison to our signature.

Studies	Published online date	PMID	Gene signature composition
Fu1 et al	2021 Mar 18	33,816,483	DCN, P4HA1
Wang et al	2021 Jul 16	34,336,848	FPR1, FCER1G
Li et al	2021 Jul 14	34,261,456	MMP9, CD74, SPP1, CXCL12, TYROBP, FCER1G, HCLS1, ARHGDIB, LAPTM5, IGF1R
Liu et al	2021 Aug 92	34,342,651	PSMC4, CXCL13, GBP2, CCL2, PPARG, CD79A, BCL10, FPR1, BMP8B, CORT, JAG2, STC2, MTNR1B, TNFRSF21
Zhang et al	2021 Aug 26	34,513,835	AMBRA1, MYC, VEGFA
Fan et al	2021 Sep 06	34,552,929	ZFP90, UHRF2, SELPLG, PLD3, PLCB4, IFNGR1, DLEU2, ATP6V1E1, ANXA5
Lei et al	2021 Sep 21	34,506,683	ALOX15B, ATG7, CBS, DPP4, EGLN1, G6PD, MUC1, MYC, PEBP1, PGD, SLC39A8, SOCS1
Xiao et al	2021 Oct 18	34,733,853	DLL1, EOMES, ERCC2, FOLR1, MEF2C, PSMA5, PTN, SPI1
Fu2 et al	2021 Dec 09	34,916,821	MYC, CLEC5A
Shi et al	2021 Dec 11	34,894,177	MYC, COL13A1, UHRF2, MT1A, ACTB, GBP1
Wu et al	2022 Jan 05	35,071,320	EGFR, CAVIN1, MXI1, SDC3,TES
Feng et al	2022 Jan 20	35,127,816	WAS, IFNGR1, PILRA, TMEM86A, CXCL16, CTNNBIP1, APOL6
Chen et al	2022 May 14	35,568,866	CSTF2, ADAR, WTAP

**Table 4:** Comparison of the nine-gene signature with previous published models in a univariate Cox analysis.

Variables	TARGET-OS				GSE21257				GSE39055			
	HR	Lower 95% CI	Upper 95% CI	*P*	HR	Lower 95% CI	Upper 95% CI	*P*	HR	Lower 95% CI	Upper 95% CI	*P*
Our study	**51.711**	**6.991**	**382.500**	**1.112E-04**	**2.631**	**1.068**	**6.481**	**3.550E-02**	**5.340**	**1.093**	**26.102**	**3.852E-02**
Fu^1^ et al	**3.131**	**1.377**	**7.119**	**6.475E-03**	1.582	0.690	3.627	2.786E-01	1.336	0.207	2.706	6.589E-01
Wang et al	**2.932**	**1.291**	**6.661**	**1.018E-02**	2.104	0.904	4.895	8.433E-02	0.581	0.163	2.074	4.033E-01
Li et al	**3.027**	**1.331**	**6.884**	**8.222E-03**	1.669	0.723	3.853	2.301E-01	3.028	0.778	11.787	1.101E-01
Liu et al	**11.642**	**3.506**	**38.652**	**6.095E-05**	0.897	0.395	2.036	7.947E-01	0.973	0.279	3.399	9.664E-01
Zhang et al	**3.940**	**1.671**	**9.290**	**1.734E-03**	1.763	0.768	4.050	1.812E-01	0.763	0.373	4.608	6.730E-01
Fan et al	**8.338**	**2.884**	**24.104**	**9.021E-05**	**3.794**	**1.506**	**9.557**	**4.680E-03**	2.058	0.124	1.911	3.015E-01
Lei et al	**5.524**	**2.099**	**14.541**	**5.377E-04**	1.083	0.476	2.462	8.492E-01	1.270	0.361	4.469	7.096E-01
Xiao et al	**6.976**	**2.638**	**18.446**	**9.020E-05**	1.828	0.789	4.236	1.594E-01	1.953	0.142	1.848	3.068E-01
Fu^2^ et al	**4.772**	**1.931**	**11.789**	**7.084E-04**	2.245	0.963	5.232	6.111E-02	1.296	0.368	4.560	6.860E-01
Shi et al	**9.765**	**3.368**	**28.314**	**2.722E-05**	1.842	0.797	4.257	1.532E-01	1.278	0.217	2.823	7.081E-01
Wu et al	**13.190**	**3.968**	**43.839**	**2.562E-05**	1.087	0.478	2.472	8.429E-01	0.520	0.133	2.037	3.476E-01
Feng et al	**4.073**	**1.722**	**9.633**	**1.388E-03**	**3.136**	**1.313**	**7.488**	**1.007E-02**	0.929	0.232	3.718	9.169E-01
Chen et al	1.728	0.815	3.662	1.538E-01	0.588	0.255	1.355	2.126E-01	1.173	0.333	4.138	8.037E-01

*CI* confidence interval, *HR* hazard ratio, *P P* value of Cox regression model. The results were in bold if *P* < 0.05.

### Identification of signature-associated biological functions and pathways in OS

**Figure 9:**
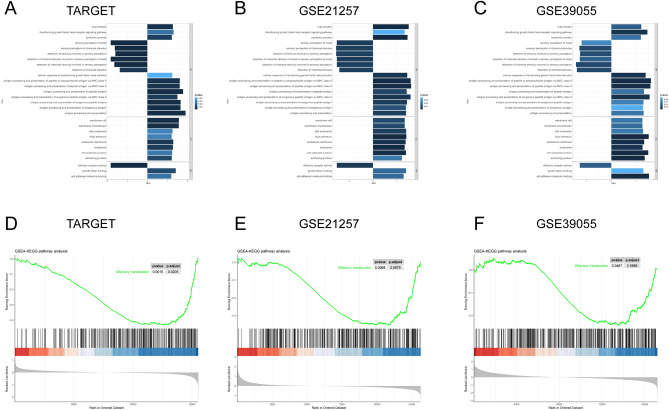
Gene set enrichment analysis of GO and KEGG pathways in OS between different signature risk score groups in the training and validation cohorts. The results of GO functions between different signature risk score groups in the TARGET (**A**), GSE21257 (**B**), and GSE39055 (**C**) datasets. The results of KEGG pathway analysis between different signature risk score groups in the TARGET (**D**), GSE21257 (**E**), and GSE39055 (**F**) datasets.

The tumor sample tissues of the three datasets were dichotomized into high- and low-risk groups according to the median of the nine-gene signature risk score. A GSEA was performed to identify the signature-associated biological functions and signaling pathways, and then the results from all datasets were merged to attain reliable results. We found that the olfactory transduction receptor signaling pathway and its related biological functions were downregulated in high-risk groups compared to low-risk groups (Fig. 9). Antigen processing- and presentation-related biological functions were also found to be upregulated in high-risk groups compared with low-risk groups. However, none of the related signaling pathways were found to be significantly correlated with different risk groups. These results indicate that the olfactory transduction receptor signaling pathway may play a role in the difference between signature-predicted outcomes.

### Signature risk scores and chemotherapy sensitivity

**Figure 10:**
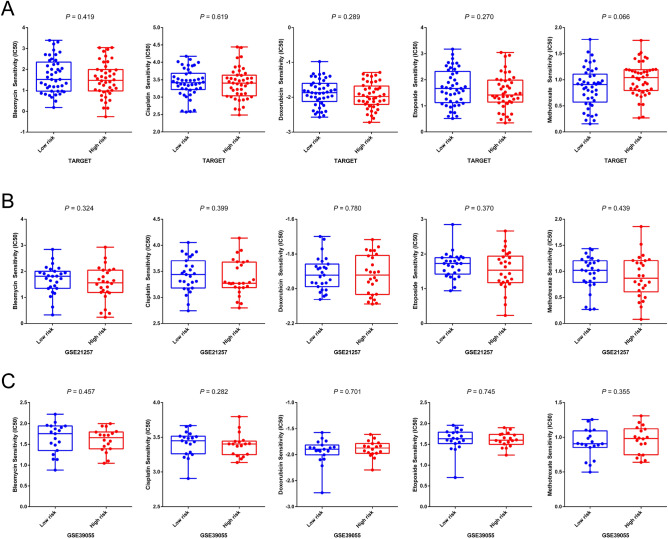
Gene signature risk score in the role of chemotherapy. (**A**) The correlation between different signature risk scores of OS patients and estimated IC50 values of bleomycin, cisplatin, doxorubicin, etoposide, and methotrexate in the TARGET (**A**), GSE21257 (**B**), and GSE39055 (**C**) datasets. The Wilcoxon signed-rank test was used to compare the estimated IC50 value of chemotherapy between groups.

To investigate whether the immune-correlated gene signature is an independent prognostic factor of OS patients, we need to adjust it based on previously reported important prognostic factors, including histologic response to chemotherapy and presence of metastases. Supplemental Figure 3 shows the results and indicates that our signature is an independent OS prognostic factor. However, not all target patients received neoadjuvant chemotherapy; thus, this group of patients lacked Huvos grade information. To validate the independence of our signature, we explored the relationship between the risk score and chemotherapy sensitivity. IC50 was calculated using the “pRRophetic” R package to predict the treatment response to chemotherapy drugs (bleomycin, cisplatin, doxorubicin, etoposide, and methotrexate). As we anticipated, there was no significant difference in the sensitivity of all drugs in the low- and high-risk groups (Fig. 10). The results are consistent with the multivariate Cox analysis. GSEA also did not reveal any biological functions or pathways responsible for chemotherapy resistance. Therefore, the overall survival difference predicted by our signature is probably due to the various backgrounds of the immune microenvironment of OS.

## Discussion

Although OS is the most common primary bone cancer in children and young adults, it is a very rare cancer, with approximately 400 new cases diagnosed annually in the USA^37^. The incidence peaks in adolescence and in old age^38^. The most common early symptom is ostalgia, which is easily confused with growing pain. The most common location of OS is in the metaphysis around the knee joint, followed by the proximal tibia and humerus. The majority of patients are therefore diagnosed with localized disease. Among the patients in the progression stage, the lungs and other bones are the most common metastatic locations^39^. The prognostic factors include tumor site, tumor size, tumor resectability, histologic response to chemotherapy, and presence of metastases^40–42^. Chemotherapy consisting of high-dose methotrexate and other drugs before and after definitive surgery has been used as the standard treatment strategy for localized osteosarcoma over the past decades^43–46^. In patients with relapsed and/or metastatic osteosarcoma, surgery and chemotherapy have some effect^3,47–49^. However, the prognosis of those patients was a median event-free survival of less than 4 months, which is unsatisfactory^50^. An increased understanding of the disease from well-annotated tissue banks through newly developed technologies has revealed its heterogeneity and molecular aberrations, which offer new insight into targeted therapy and immunotherapy.

Recently, immunotherapy has facilitated a revolution in many kinds of solid malignant tumor treatments. Although its efficiency is not so high, it can lead to considerable survival benefits among responders^51^. Osteosarcoma humanized in vitro and humanized mouse models with similar tumor microenvironments in several previous studies have indicated that immunotherapy targeting the tumor microenvironment may be a promising strategy for osteosarcoma treatment^11–13^. Studies aiming at finding the precise immune response population have also suggested that the tumor immune microenvironment is the critical factor^52^. Therefore, it is essential to better understand and characterize the immune status of OS for the precise prediction of the immune response and the development of immunotherapy. The OS immune microenvironment consists of a network of immune cells that function as ideal grounds for tumor proliferation and progression^15,53^.

Our study first found that M0 macrophages were the dominant immune cells in the TARGET and GEO databases using the CIBERSORT algorithm. However, the direct association between M0 macrophage infiltration and the overall survival of OS patients was not consistent in different independent datasets (Supplemental Figure 4). Based on the hypothesis that the dominant immune cells play the most important role in the OS microenvironment, we screened for genes associated with M0 macrophages and built a new nine-gene signature for OS prognosis using clinical information from the TARGET dataset. In addition, the prognostic signature was proven to be effective and reliable in validation cohorts consisting of two independent GEO datasets. Previously published prognostic gene signatures were compared with the newly discovered gene signature using publicly available datasets. The results showed that no signatures besides ours offer a precise prediction value of OS survival in all datasets, indicating the priority of the nine-gene signature. Multivariate Cox regression models indicate that the signature risk score is an independent prognostic factor for OS patients regardless of age, sex, or Huvos grade in all datasets. Furthermore, the association between the signature risk score and chemotherapy sensitivity also showed that there was no significant difference in the sensitivity of all drugs between the low- and high-risk groups. Therefore, the different outcomes predicted by our signature are probably due to the various immune microenvironments of OS. To our knowledge, this is the first time a study has been conducted on the dominant infiltrating immune cell profiles in several datasets to find similarities between OS tumor immune microenvironments. As we expected, a robust gene signature was generated based on the discovered similarities.

M0 macrophages are slightly elongated unstimulated macrophages, which are considered to be theoretically inactivated^54^. Previous studies have suggested that M0 macrophages are associated with poor prognosis and metastatic disease in lung adenocarcinoma^55,56^, hepatocellular carcinoma^57^, osteosarcoma^53^, pancreatic adenocarcinoma^58^, melanoma^59^, colorectal cancer^60^, gastric cancer^61^, and glioblastoma^62^. Recent studies have shown that new tumor-infiltrated M0 macrophages induce pancreatic cancer cell death via TNF-α secretion, while M1, M2, and tumor-associated macrophages do not harbor antitumorigenic activities^63^. However, mesenchymal stem cells could activate STAT6 and induce M2 polarization through disease progression, leading to anti-inflammatory functions^64^, which may explain the relationship between high M0 macrophage infiltration and poor prognosis.

Our prognostic signature consisted of nine genes (Table 2): CPE, FHL5, GBP1, GNLY, GPR82, IL18RAP, LILRA2, NDRG4, and PLB1. In our research, CPE was determined to be an unfavorable biomarker for OS prognosis, while PLB1 exhibited protective effects in OS (Supplemental Figure 5). CPE encodes carboxypeptidase E, which belongs to the carboxypeptidase family and is reported to be involved in the biosynthesis of hormone and neurotransmitter peptides and to play nonenzymatic roles in the endocrine and nervous systems^65^. However, previous studies have also proven that CPE plays various roles in various cancers^66^. Its N-terminal truncated protein has been found to be expressed in multiple cancers, regulate metastatic gene expression, and encompass different signaling pathways^67^. CPE has been reported to reduce aerobic glycolysis and migration in glioblastoma cells^68^. In addition, CPE may promote migration and invasiveness in various other cancers, such as lung cancer^69^, pancreatic cancer^70^, and cervical cancer^71^. In osteosarcoma, Fan et al. found that CPE-variant proteins increase invasiveness and promote epithelial-mesenchymal transition through the activation of the Wnt pathway in OS cells^72^. CPE also modulates immunity. Bar et al. found that it regulates immune homeostasis and inhibits inflammation^73^. Sanjay also reported that it enhances the innate immunity of the male reproductive tract^74^. Therefore, whether CPE plays a role in cancer immunity needs to be discussed further. PLB1 has seldom been studied in cancer. Its rearrangement with ALK has been found in lung cancer^75^, which indicates a sensitivity to targeted therapy^76^. Lin et al. reported that overexpressed PLB1 antigens cause high infiltration of immune cells and a favorable prognosis in glioblastoma patients^77^. PLB1 is commonly discussed in yeast and was found to be essential for the survival and virulence of Cryptococcus^78^ and *Candida albicans*^79^.

Recently, with the appearance of advanced bioinformatics algorithms, numerous gene signatures have been built from public online datasets. However, the universalism of those signatures was largely undetected or only detected in one independent cohort. Regarding the limitations of previous findings, the nine-gene signatures were summarized from the generality of several datasets. To determine the priority of our hypothesis, we included previous potential survival-related OS signatures from recent studies published in journals with a high influence and good reputation for comparison^80–92^. As we expected, after validating them in the training and validation cohorts, the nine-gene signature was the only signature showing a consistent predictive value for OS survival. Among the previous studies, the signatures by Fan et al. and Feng et al. could accurately predict the prognosis of OS in the TARGET and GSE21257 datasets. However, in the GSE39055 dataset, the lines of different groups predicted by Feng’s signature in the K‒M plot were very close, while the survival difference of groups predicted by Fan’s signature showed a reverse tendency compared with other datasets.

A GSEA of GO and KEGG pathways found that antigen processing- and presentation-related biological functions and olfactory transduction receptor signaling pathways play important roles in signature functioning. Numerous studies have proven that antigen processing- and presentation-related biological functions are key functions in the cancer immune response process and are mainly carried out by dendritic cells, macrophages and B cells^93–95^. These results indicate the important role of the three antigen-presenting cells and cancer immunity in the prognosis of OS, which is consistent with our hypothesis. Regarding the olfactory transduction receptor signaling pathway, a previous study reported that olfactory receptors provide innate and adaptive immune responses during the virus entry process^96^. Orecchioni et al. recently found that immune cells, such as vascular macrophages, express olfactory receptors, which induce interleukin-1β secretion, leading to inflammation^97^. Nevertheless, there have not been any studies that have reported any association between the olfactory transduction receptor signaling pathway and cancer immunity thus far.

Our analysis also showed the homogeneity and heterogeneity of the osteosarcoma immune microenvironment. M0 macrophages were the dominant infiltrating immune cell type in all datasets. In addition, the infiltration of M2 macrophages, which have been reported to play an immune repressive role in cancer^98^, was similar to that of M0 macrophages in the three datasets, except for the GSE39055 dataset. There were also several infiltrated CD8 + T cells in the TARGET and GSE16091 datasets. Other immune cell infiltration levels were quite low. The above results may partly explain the unsatisfactory response to current immunotherapy in osteosarcoma. Most of the immune microenvironment of osteosarcoma tissues consists of immune repressive M2 macrophages or nonfunctional M0 macrophages. Patients with tumor tissues infiltrated with several CD8 + T cells may receive survival benefits from immunotherapy. Given the large proportion of M0 and M2 macrophages in osteosarcoma immune cells, inducing M0 or M2 macrophages toward the M1 phenotype to promote the antitumor immune response may be a promising treatment strategy for OS patients.

There are several limitations in the present study. First, the prognostic signature was constructed from public online retrospective data with relatively small sample sizes. It may be improved by future OS studies with larger sample sizes. Second, the predictive value of the signature needs to be confirmed by future prospective studies. Third, the mechanism and function of a high infiltration of M0 macrophages in the OS microenvironment is still unclear and needs to be clarified in the future. Fourth, many prognostic factors were unavailable in the datasets used, and as such, the independence of the signature could not be fully determined. In addition, there are no wet-lab experimental data supporting the roles of signature genes and the olfactory transduction receptor signaling pathway in immune infiltration and other biological processes. Therefore, further research is needed to investigate the mechanisms.

## Conclusions

In the present study, we built a new and robust nine-gene signature based on the hypothesis that the dominant infiltrated immune cells play the most important role in OS progression. The newly defined gene signature was found to be significantly associated with OS prognosis in all datasets. In addition, we proved the advantage of the signature by comparing it to previously published signatures. Antigen processing- and presentation-related biological functions and the olfactory transduction receptor signaling pathway were found to be associated with the signature risk score. The potential role and mechanism of the olfactory transduction receptor signaling pathway and M0 macrophages in OS should be evaluated in the future.

### Supplementary Information

Supplementary Information.

## Data Availability

All datasets analyzed in the present study are open access. These data can be found on the following websites: TARGET-OS (https://ocg.cancer.gov/programs/target/projects/osteosarcoma) and GEO database (https://www.ncbi.nlm.nih.gov/geo/query/acc.cgi?acc=GSE21257; https://www.ncbi.nlm.nih.gov/geo/query/acc.cgi?acc=GSE16091; https://www.ncbi.nlm.nih.gov/geo/query/acc.cgi?acc=GSE39055).

## Abbreviations

OS: Osteosarcoma
ROC: Receiver operating characteristic curve
I/D AUC: Incident/dynamic area under the curve
Cor: Correlation coefficient
HR: Hazard ratio
CI: Confidence interval

## References

[1] Siegel RL, Miller KD, Fuchs HE, Jemal A (2021). Cancer statistics, 2021. CA Cancer J. Clin..

[2] Miller KD, Fidler-Benaoudia M, Keegan TH, Hipp HS, Jemal A (2020). Cancer statistics for adolescents and young adults, 2020. CA Cancer J. Clin..

[3] Daw NC, Chou AJ, Jaffe N, Rao BN, Billups CA (2015). Recurrent osteosarcoma with a single pulmonary metastasis: A multi-institutional review. Br. J. Cancer.

[4] Lewis IJ, Nooij MA, Whelan J, Sydes MR, Grimer R (2007). Improvement in histologic response but not survival in osteosarcoma patients treated with intensified chemotherapy: A randomized phase III trial of the European osteosarcoma intergroup. J. Natl. Cancer Inst..

[5] Lahr CA, Landgraf M, Wagner F, Cipitria A, Moreno-Jimenez I (2022). A humanised rat model of osteosarcoma reveals ultrastructural differences between bone and mineralised tumour tissue. Bone.

[6] Kundu B, Bastos ARF, Brancato V, Cerqueira MT, Oliveira JM (2019). Mechanical property of hydrogels and the presence of adipose stem cells in tumor stroma affect spheroid formation in the 3D osteosarcoma model. ACS Appl. Mater. Interfaces.

[7] Wang ML, Xu NY, Tang RZ, Liu XQ (2022). A 3D-printed scaffold-based osteosarcoma model allows to investigate tumor phenotypes and pathogenesis in an in vitro bone-mimicking niche. Mater. Today Bio..

[8] de Nigris F, Crudele V, Giovane A, Casamassimi A, Giordano A (2010). CXCR4/YY1 inhibition impairs VEGF network and angiogenesis during malignancy. Proc. Natl. Acad. Sci. USA.

[9] Wagner F, Holzapfel BM, Martine LC, McGovern J, Lahr CA (2019). A humanized bone microenvironment uncovers HIF2 alpha as a latent marker for osteosarcoma. Acta Biomater..

[10] Monteiro CF, Custodio CA, Mano JF (2021). Bioengineering a humanized 3D tri-culture osteosarcoma model to assess tumor invasiveness and therapy response. Acta Biomater..

[11] Belisario DC, Akman M, Godel M, Campani V, Patrizio MP (2020). ABCA1/ABCB1 ratio determines chemo- and immune-sensitivity in human osteosarcoma. Cells.

[12] Kurelac I, Abarrategi A, Ragazzi M, Iommarini L, Umesh Ganesh N (2019). A humanized bone niche model reveals bone tissue preservation upon targeting mitochondrial complex I in pseudo-orthotopic osteosarcoma. J. Clin. Med..

[13] Zheng B, Ren T, Huang Y, Sun K, Wang S (2018). PD-1 axis expression in musculoskeletal tumors and antitumor effect of nivolumab in osteosarcoma model of humanized mouse. J. Hematol. Oncol..

[14] Cappariello A, Rucci N (2019). Tumour-derived extracellular vesicles (EVs): A dangerous, "message in a bottle" for bone. Int. J. Mol. Sci..

[15] Chen C, Xie L, Ren T, Huang Y, Xu J (2021). Immunotherapy for osteosarcoma: Fundamental mechanism, rationale, and recent breakthroughs. Cancer Lett..

[16] Wolf-Dennen K, Gordon N, Kleinerman ES (2020). Exosomal communication by metastatic osteosarcoma cells modulates alveolar macrophages to an M2 tumor-promoting phenotype and inhibits tumoricidal functions. Oncoimmunology.

[17] Koirala P, Roth ME, Gill J, Chinai JM, Ewart MR (2016). HHLA2, a member of the B7 family, is expressed in human osteosarcoma and is associated with metastases and worse survival. Sci. Rep..

[18] Koirala P, Roth ME, Gill J, Piperdi S, Chinai JM (2016). Immune infiltration and PD-L1 expression in the tumor microenvironment are prognostic in osteosarcoma. Sci. Rep..

[19] Gomez-Brouchet A, Illac C, Gilhodes J, Bouvier C, Aubert S (2017). CD163-positive tumor-associated macrophages and CD8-positive cytotoxic lymphocytes are powerful diagnostic markers for the therapeutic stratification of osteosarcoma patients: An immunohistochemical analysis of the biopsies fromthe French OS2006 phase 3 trial. Oncoimmunology.

[20] Dumars C, Ngyuen JM, Gaultier A, Lanel R, Corradini N (2016). Dysregulation of macrophage polarization is associated with the metastatic process in osteosarcoma. Oncotarget.

[21] Buddingh EP, Kuijjer ML, Duim RA, Burger H, Agelopoulos K (2011). Tumor-infiltrating macrophages are associated with metastasis suppression in high-grade osteosarcoma: A rationale for treatment with macrophage activating agents. Clin. Cancer Res..

[22] Corre I, Verrecchia F, Crenn V, Redini F, Trichet V (2020). The osteosarcoma microenvironment: A complex but targetable ecosystem. Cells.

[23] Wang J, Zhang H, Sun X, Wang X, Ren T (2020). Exosomal PD-L1 and N-cadherin predict pulmonary metastasis progression for osteosarcoma patients. J. Nanobiotechnol..

[24] Troyer RM, Ruby CE, Goodall CP, Yang L, Maier CS (2017). Exosomes from osteosarcoma and normal osteoblast differ in proteomic cargo and immunomodulatory effects on T cells. Exp. Cell Res..

[25] Zhou Q, Xian M, Xiang S, Xiang D, Shao X (2017). All-trans retinoic acid prevents osteosarcoma metastasis by inhibiting M2 polarization of tumor-associated macrophages. Cancer Immunol. Res..

[26] Li T, Fan J, Wang B, Traugh N, Chen Q (2017). TIMER: A web server for comprehensive analysis of tumor-infiltrating immune cells. Cancer Res..

[27] Li T, Fu J, Zeng Z, Cohen D, Li J (2020). TIMER2.0 for analysis of tumor-infiltrating immune cells. Nucl. Acids Res..

[28] Gong L, Jia L (2023). ABCC8 is correlated with immune cell infiltration and overall survival in lower grade glioma. BIOCELL.

[29] Newman AM, Steen CB, Liu CL, Gentles AJ, Chaudhuri AA (2019). Determining cell type abundance and expression from bulk tissues with digital cytometry. Nat. Biotechnol..

[30] Thorsson V, Gibbs DL, Brown SD, Wolf D, Bortone DS (2018). The immune landscape of cancer. Immunity.

[31] Newman AM, Liu CL, Green MR, Gentles AJ, Feng W (2015). Robust enumeration of cell subsets from tissue expression profiles. Nat. Methods.

[32] Yoshihara K, Shahmoradgoli M, Martinez E, Vegesna R, Kim H (2013). Inferring tumour purity and stromal and immune cell admixture from expression data. Nat. Commun..

[33] Stanley TD, Doucouliagos H, Ioannidis JPA (2022). Retrospective median power, false positive meta-analysis and large-scale replication. Res. Synth. Methods.

[34] Heagerty PJ, Lumley T, Pepe MS (2000). Time-dependent ROC curves for censored survival data and a diagnostic marker. Biometrics.

[35] Geeleher P, Cox N, Huang RS (2014). pRRophetic: An R package for prediction of clinical chemotherapeutic response from tumor gene expression levels. PLoS One.

[36] Yang W, Soares J, Greninger P, Edelman EJ, Lightfoot H (2013). Genomics of drug sensitivity in cancer (GDSC): A resource for therapeutic biomarker discovery in cancer cells. Nucl. Acids Res..

[37] Cole S, Gianferante DM, Zhu B, Mirabello L (2022). Osteosarcoma: A surveillance, epidemiology, and end results program-based analysis from 1975 to 2017. Cancer.

[38] Gill J, Gorlick R (2021). Advancing therapy for osteosarcoma. Nat. Rev. Clin. Oncol..

[39] Bielack SS, Kempf-Bielack B, Delling G, Exner GU, Flege S (2002). Prognostic factors in high-grade osteosarcoma of the extremities or trunk: An analysis of 1702 patients treated on neoadjuvant cooperative osteosarcoma study group protocols. J. Clin. Oncol..

[40] Harris MB, Gieser P, Goorin AM, Ayala A, Shochat SJ (1998). Treatment of metastatic osteosarcoma at diagnosis: A pediatric oncology group study. J. Clin. Oncol..

[41] Pakos EE, Nearchou AD, Grimer RJ, Koumoullis HD, Abudu A (2009). Prognostic factors and outcomes for osteosarcoma: An international collaboration. Eur. J. Cancer.

[42] Bacci G, Rocca M, Salone M, Balladelli A, Ferrari S (2008). High grade osteosarcoma of the extremities with lung metastases at presentation: treatment with neoadjuvant chemotherapy and simultaneous resection of primary and metastatic lesions. J. Surg. Oncol..

[43] Gaspar N, Occean BV, Pacquement H, Bompas E, Bouvier C (2018). Results of methotrexate-etoposide-ifosfamide based regimen (M-EI) in osteosarcoma patients included in the French OS2006/sarcome-09 study. Eur. J. Cancer.

[44] Marina NM, Smeland S, Bielack SS, Bernstein M, Jovic G (2016). Comparison of MAPIE versus MAP in patients with a poor response to preoperative chemotherapy for newly diagnosed high-grade osteosarcoma (EURAMOS-1): An open-label, international, randomised controlled trial. Lancet Oncol..

[45] Ferrari S, Ruggieri P, Cefalo G, Tamburini A, Capanna R (2012). Neoadjuvant chemotherapy with methotrexate, cisplatin, and doxorubicin with or without ifosfamide in nonmetastatic osteosarcoma of the extremity: An Italian sarcoma group trial ISG/OS-1. J. Clin. Oncol..

[46] Meyers PA, Schwartz CL, Krailo MD, Healey JH, Bernstein ML (2008). Osteosarcoma: The addition of muramyl tripeptide to chemotherapy improves overall survival–a report from the Children's oncology group. J. Clin. Oncol..

[47] Buddingh EP, Anninga JK, Versteegh MI, Taminiau AH, Egeler RM (2010). Prognostic factors in pulmonary metastasized high-grade osteosarcoma. Pediatr. Blood Cancer.

[48] Palmerini E, Jones RL, Marchesi E, Paioli A, Cesari M (2016). Gemcitabine and docetaxel in relapsed and unresectable high-grade osteosarcoma and spindle cell sarcoma of bone. BMC Cancer.

[49] Berrak SG, Pearson M, Berberoglu S, Ilhan IE, Jaffe N (2005). High-dose ifosfamide in relapsed pediatric osteosarcoma: Therapeutic effects and renal toxicity. Pediatr. Blood Cancer.

[50] Lagmay JP, Krailo MD, Dang H, Kim A, Hawkins DS (2016). Outcome of patients with recurrent osteosarcoma enrolled in seven phase II trials through children's cancer group, pediatric oncology group, and children's oncology group: Learning from the past to move forward. J. Clin. Oncol.

[51] Yahiro K, Matsumoto Y (2021). Immunotherapy for osteosarcoma. Hum. Vaccin. Immunother..

[52] Inthagard J, Edwards J, Roseweir AK (2019). Immunotherapy: Enhancing the efficacy of this promising therapeutic in multiple cancers. Clin. Sci. (Lond.).

[53] Le T, Su S, Shahriyari L (2021). Immune classification of osteosarcoma. Math. Biosci. Eng..

[54] Wosik J, Chen W, Qin K, Ghobrial RM, Kubiak JZ (2018). Magnetic field changes macrophage phenotype. Biophys. J..

[55] Yi M, Li A, Zhou L, Chu Q, Luo S (2021). Immune signature-based risk stratification and prediction of immune checkpoint inhibitor's efficacy for lung adenocarcinoma. Cancer Immunol. Immunother..

[56] Mo Z, Yu L, Cao Z, Hu H, Luo S (2020). Identification of a hypoxia-associated signature for lung adenocarcinoma. Front. Genet..

[57] Farha M, Jairath NK, Lawrence TS, El Naqa I (2020). Characterization of the tumor immune microenvironment identifies M0 macrophage-enriched cluster as a poor prognostic factor in hepatocellular carcinoma. JCO Clin. Cancer Inform..

[58] Xu F, Zhang Z, Yuan M, Zhao Y, Zhou Y (2021). M6A regulatory genes play an important role in the prognosis, progression and immune microenvironment of pancreatic adenocarcinoma. Cancer Invest..

[59] Jairath NK, Farha MW, Jairath R, Harms PW, Tsoi LC (2020). Prognostic value of intratumoral lymphocyte-to-monocyte ratio and M0 macrophage enrichment in tumor immune microenvironment of melanoma. Melanoma Manag.

[60] Ge P, Wang W, Li L, Zhang G, Gao Z (2019). Profiles of immune cell infiltration and immune-related genes in the tumor microenvironment of colorectal cancer. Biomed. Pharmacother..

[61] Nie K, Zheng Z, Wen Y, Shi L, Xu S (2020). Construction and validation of a TP53-associated immune prognostic model for gastric cancer. Genomics.

[62] Gabrusiewicz K, Rodriguez B, Wei J, Hashimoto Y, Healy LM (2016). Glioblastoma-infiltrated innate immune cells resemble M0 macrophage phenotype. JCI Insight.

[63] Tekin C, Aberson HL, Bijlsma MF, Spek CA (2020). Early macrophage infiltrates impair pancreatic cancer cell growth by TNF-alpha secretion. BMC Cancer.

[64] Li Y, Sheng Q, Zhang C, Han C, Bai H (2021). STAT6 up-regulation amplifies M2 macrophage anti-inflammatory capacity through mesenchymal stem cells. Int. Immunopharmacol..

[65] Fricker LD (1988). Carboxypeptidase E. Annu. Rev. Physiol..

[66] Cawley NX, Wetsel WC, Murthy SR, Park JJ, Pacak K (2012). New roles of carboxypeptidase E in endocrine and neural function and cancer. Endocr. Rev..

[67] Yang X, Lou H, Chen YT, Huang SF, Loh YP (2019). A novel 40kDa N-terminal truncated carboxypeptidase E splice variant: cloning, cDNA sequence analysis and role in regulation of metastatic genes in human cancers. Genes Cancer.

[68] Ilina EI, Armento A, Sanchez LG, Reichlmeir M, Braun Y (2017). Effects of soluble CPE on glioma cell migration are associated with mTOR activation and enhanced glucose flux. Oncotarget.

[69] Sun J, Meng D, Yu T, Li F, Zhang G (2020). N-terminal truncated carboxypeptidase E represses E-cadherin expression in lung cancer by stabilizing the Snail-HDAC complex. Am. J. Cancer Res..

[70] Lou H, Loh YP (2021). Silencing of Carboxypeptidase E expression inhibits proliferation and invasion of Panc-1 pancreatic cancer cells. F1000Res.

[71] Shen HW, Tan JF, Shang JH, Hou MZ, Liu J (2016). CPE overexpression is correlated with pelvic lymph node metastasis and poor prognosis in patients with early-stage cervical cancer. Arch. Gynecol. Obstet..

[72] Fan S, Gao X, Chen P, Li X (2019). Carboxypeptidase E-DeltaN promotes migration, invasiveness, and epithelial-mesenchymal transition of human osteosarcoma cells via the Wnt-beta-catenin pathway. Biochem. Cell Biol..

[73] Bar F, Foh B, Pagel R, Schroder T, Schlichting H (2014). Carboxypeptidase E modulates intestinal immune homeostasis and protects against experimental colitis in mice. PLoS One.

[74] Kumar S, Tomar AK, Singh S, Gill K, Dey S (2014). Heparin binding carboxypeptidase E protein exhibits antibacterial activity in human semen. Int. J. Biol. Macromol..

[75] Wang B, Chen R, Wang C, Chen H, Zhong D (2021). PLB1-ALK: A novel head-to-head fusion gene identified by next-generation sequencing in a lung adenocarcinoma patient. Lung Cancer.

[76] Wang S, Wu X, Zhao J, Chen H, Zhang Z (2021). Next-generation sequencing identified a novel crizotinib-sensitive PLB1-ALK rearrangement in lung large-cell neuroendocrine carcinoma. Clin. Lung Cancer.

[77] Lin H, Wang K, Xiong Y, Zhou L, Yang Y (2022). Identification of tumor antigens and immune subtypes of glioblastoma for mRNA vaccine development. Front. Immunol..

[78] Noverr MC, Cox GM, Perfect JR, Huffnagle GB (2003). Role of PLB1 in pulmonary inflammation and cryptococcal eicosanoid production. Infect. Immun..

[79] Mukherjee PK, Seshan KR, Leidich SD, Chandra J, Cole GT (2001). Reintroduction of the PLB1 gene into *Candida albicans* restores virulence in vivo. Microbiology (Reading).

[80] Chen S, Zeng J, Huang L, Peng Y, Yan Z (2022). RNA adenosine modifications related to prognosis and immune infiltration in osteosarcoma. J. Transl. Med..

[81] Feng X, Zhao Z, Zhao Y, Song Z, Ma Y (2021). Development of personalized signature based on the immune landscape to predict the prognosis of osteosarcoma and the response to immunotherapy and targeted therapy. Front. Mol. Biosci..

[82] Wu F, Xu J, Jin M, Jiang X, Li J (2021). Development and verification of a hypoxic gene signature for predicting prognosis, immune microenvironment, and chemosensitivity for osteosarcoma. Front. Mol. Biosci..

[83] Fu Y, He G, Liu Z, Wang J, Zhang Z (2021). Exploration and validation of a novel inflammatory response-associated gene signature to predict osteosarcoma prognosis and immune infiltration. J. Inflamm. Res..

[84] Shi D, Mu S, Pu F, Liu J, Zhong B (2022). Integrative analysis of immune-related multi-omics profiles identifies distinct prognosis and tumor microenvironment patterns in osteosarcoma. Mol. Oncol..

[85] Xiao B, Liu L, Chen Z, Li A, Xia Y (2021). A Novel overall survival prediction signature based on cancer stem cell-related genes in osteosarcoma. Front. Cell Dev. Biol..

[86] Fan L, Ru J, Liu T, Ma C (2021). Identification of a novel prognostic gene signature from the immune cell infiltration landscape of osteosarcoma. Front. Cell Dev. Biol..

[87] Zhang J, Ding R, Wu T, Jia J, Cheng X (2021). Autophagy-related genes and long noncoding RNAs signatures as predictive biomarkers for osteosarcoma survival. Front. Cell Dev. Biol..

[88] Lei T, Qian H, Lei P, Hu Y (2021). Ferroptosis-related gene signature associates with immunity and predicts prognosis accurately in patients with osteosarcoma. Cancer Sci..

[89] Liu W, Xie X, Qi Y, Wu J (2021). Exploration of immune-related gene expression in osteosarcoma and association with outcomes. JAMA Netw. Open.

[90] Wang X, Wang L, Xu W, Wang X, Ke D (2021). Classification of osteosarcoma based on immunogenomic profiling. Front. Cell Dev. Biol..

[91] Li W, Ding Z, Wang D, Li C, Pan Y (2021). Ten-gene signature reveals the significance of clinical prognosis and immuno-correlation of osteosarcoma and study on novel skeleton inhibitors regarding MMP9. Cancer Cell Int..

[92] Fu Y, Bao Q, Liu Z, He G, Wen J (2021). Development and validation of a hypoxia-associated prognostic signature related to osteosarcoma metastasis and immune infiltration. Front. Cell Dev. Biol..

[93] DeNardo DG, Ruffell B (2019). Macrophages as regulators of tumour immunity and immunotherapy. Nat. Rev. Immunol..

[94] Mitchell D, Chintala S, Dey M (2018). Plasmacytoid dendritic cell in immunity and cancer. J. Neuroimmunol..

[95] Gardner A, Ruffell B (2016). Dendritic cells and cancer immunity. Trends Immunol..

[96] Durrant DM, Ghosh S, Klein RS (2016). The olfactory bulb: An immunosensory effector organ during neurotropic viral infections. ACS Chem. Neurosci..

[97] Orecchioni M, Kobiyama K, Winkels H, Ghosheh Y, McArdle S (2022). Olfactory receptor 2 in vascular macrophages drives atherosclerosis by NLRP3-dependent IL-1 production. Science.

[98] Xia Y, Rao L, Yao H, Wang Z, Ning P (2020). Engineering macrophages for cancer immunotherapy and drug delivery. Adv. Mater..

