# Supplementary material for: New gene signature from the dominant infiltration immune cell type in osteosarcoma predicts overall survival
Source: Sci Rep. 2023 Oct 25;13:18271. doi: 10.1038/s41598-023-45566-6 (PMC10600156; doi:10.1038/s41598-023-45566-6)
Supplement: Supplementary file 1 — Supplementary Information. [file 41598_2023_45566_MOESM1_ESM.docx]

Supplemental Figure 1. Kaplan‒Meier overall survival analysis in OS patients from the TARGET, GSE21257, GSE16091, and GSE39055 datasets.


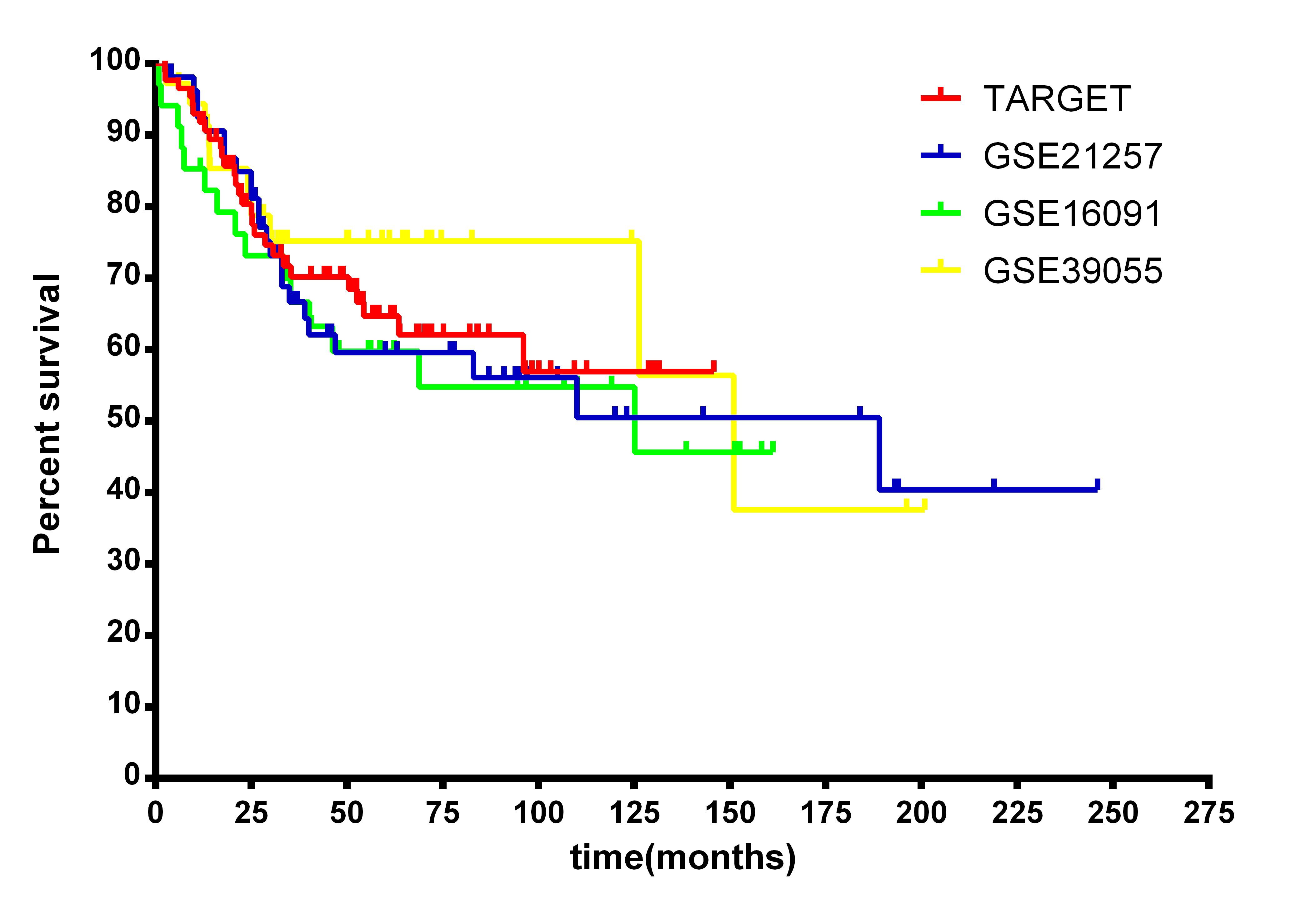


Supplemental Figure 2. Association between immune cell infiltration levels and the signature risk score of OS. Association between immune cell infiltration levels and signature risk score of OS in the TARGET (A), GSE21257 (B), and GSE39055 (C) datasets.


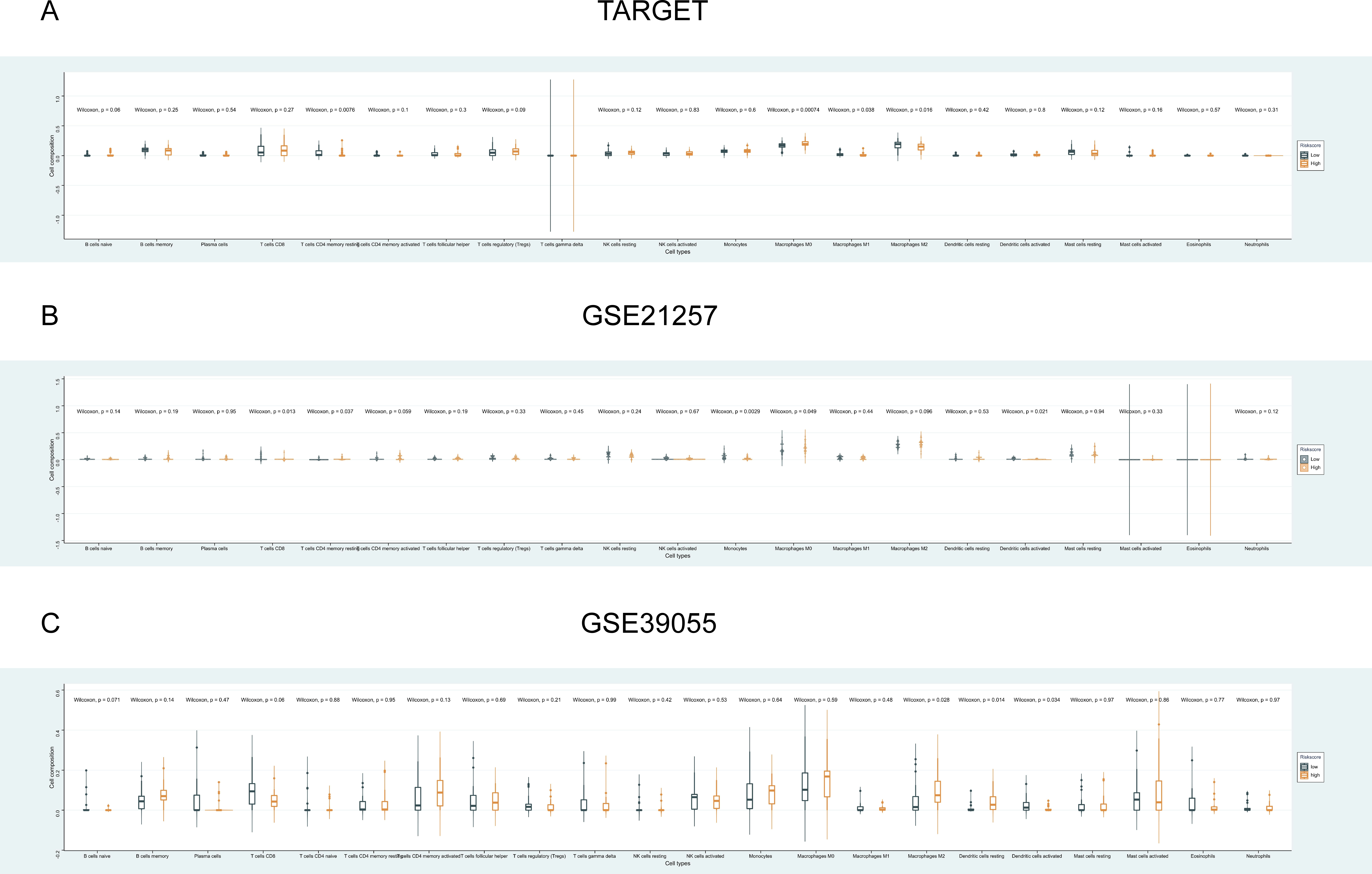


Supplemental Figure 3. Forest plot showing the results of multiple factors in the Cox regression analysis of the gene signature risk score with other clinical characteristics in OS of the TARGET dataset.


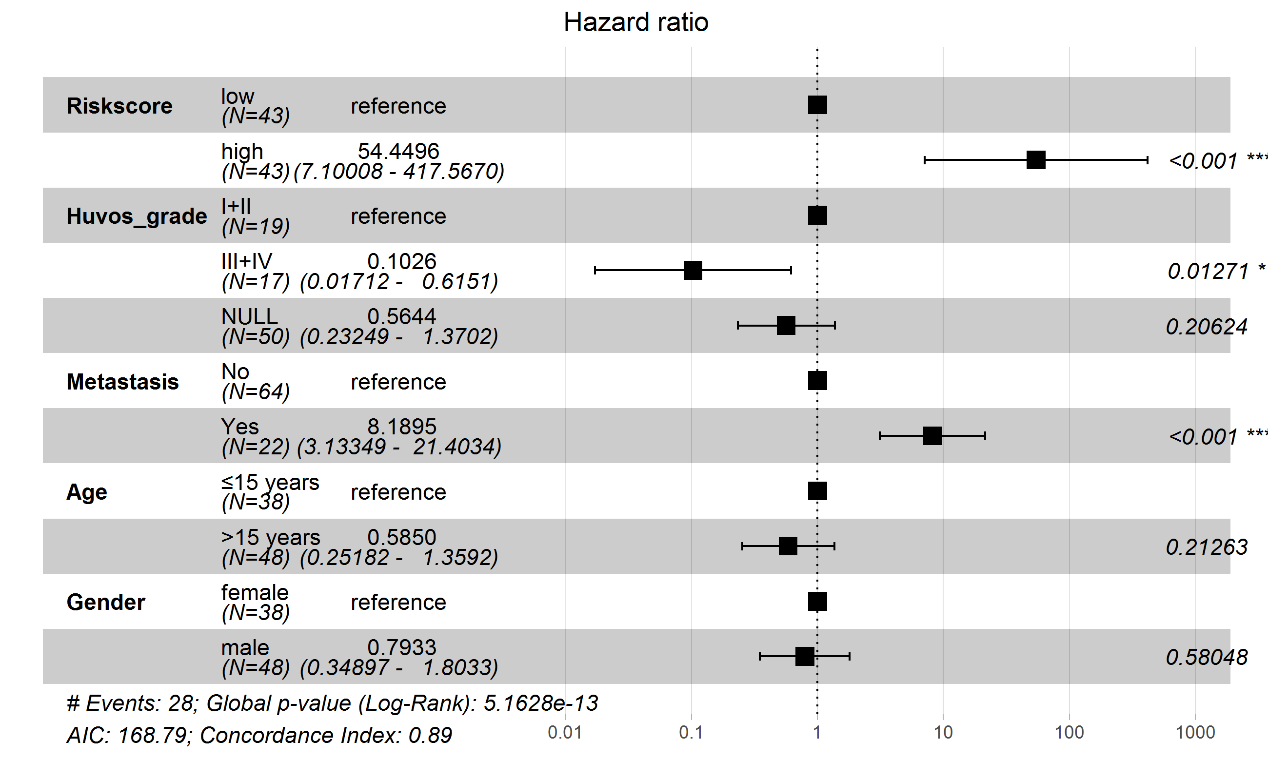


Supplemental Figure 4. Kaplan‒Meier overall survival analysis of M0 macrophage infiltration levels in OS patients in the TARGET (A), GSE21257 (B), GSE16091 (C), and GSE39055 datasets (D). Only the tumor samples with a P value <0.05 after CIBERSORT estimation were included.


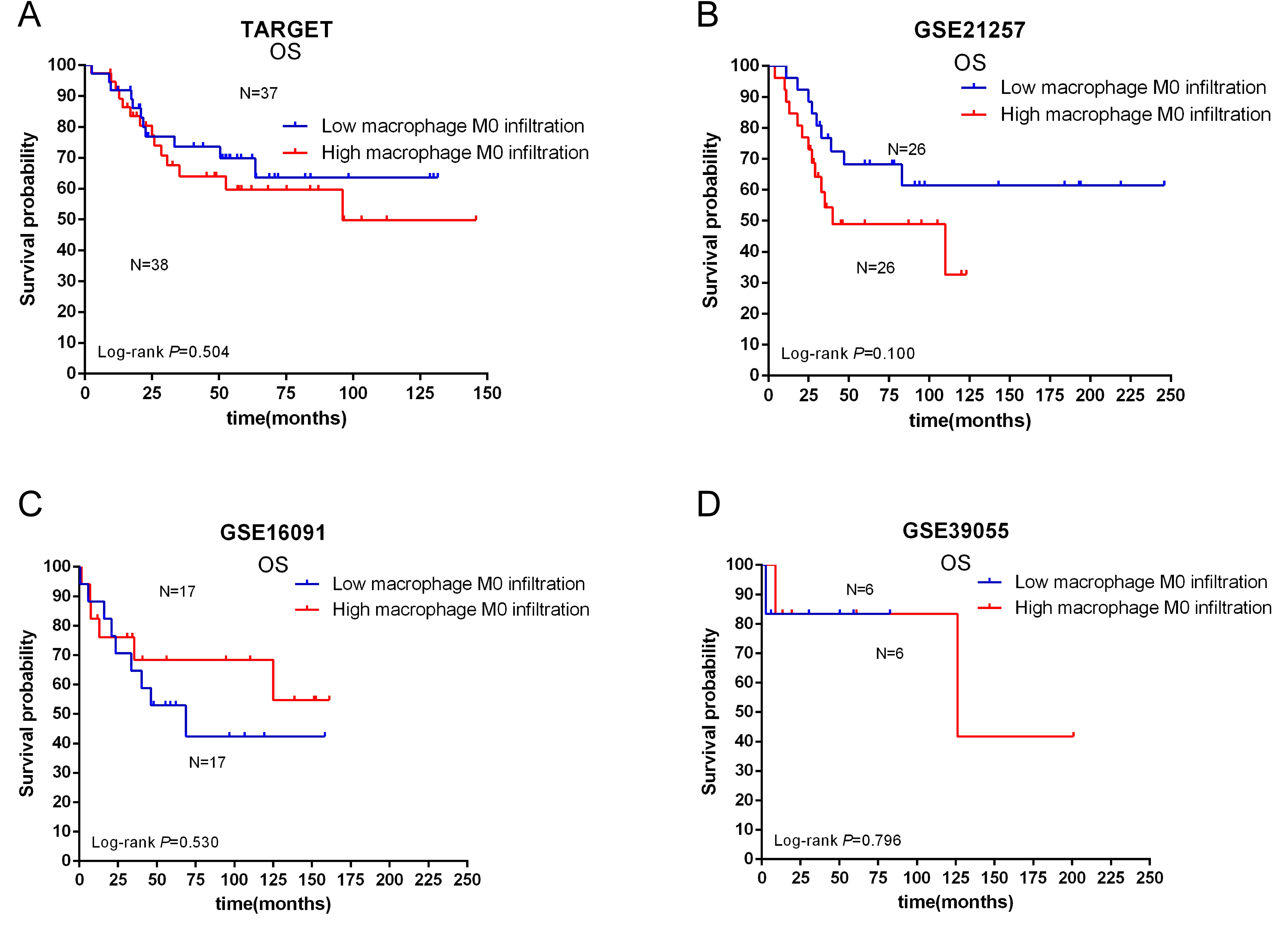


Supplemental Figure 5. Survival analysis of the nine genes in the present signature using the Kaplan–Meier estimator in the TARGET, GSE21257, and GSE39055 datasets.


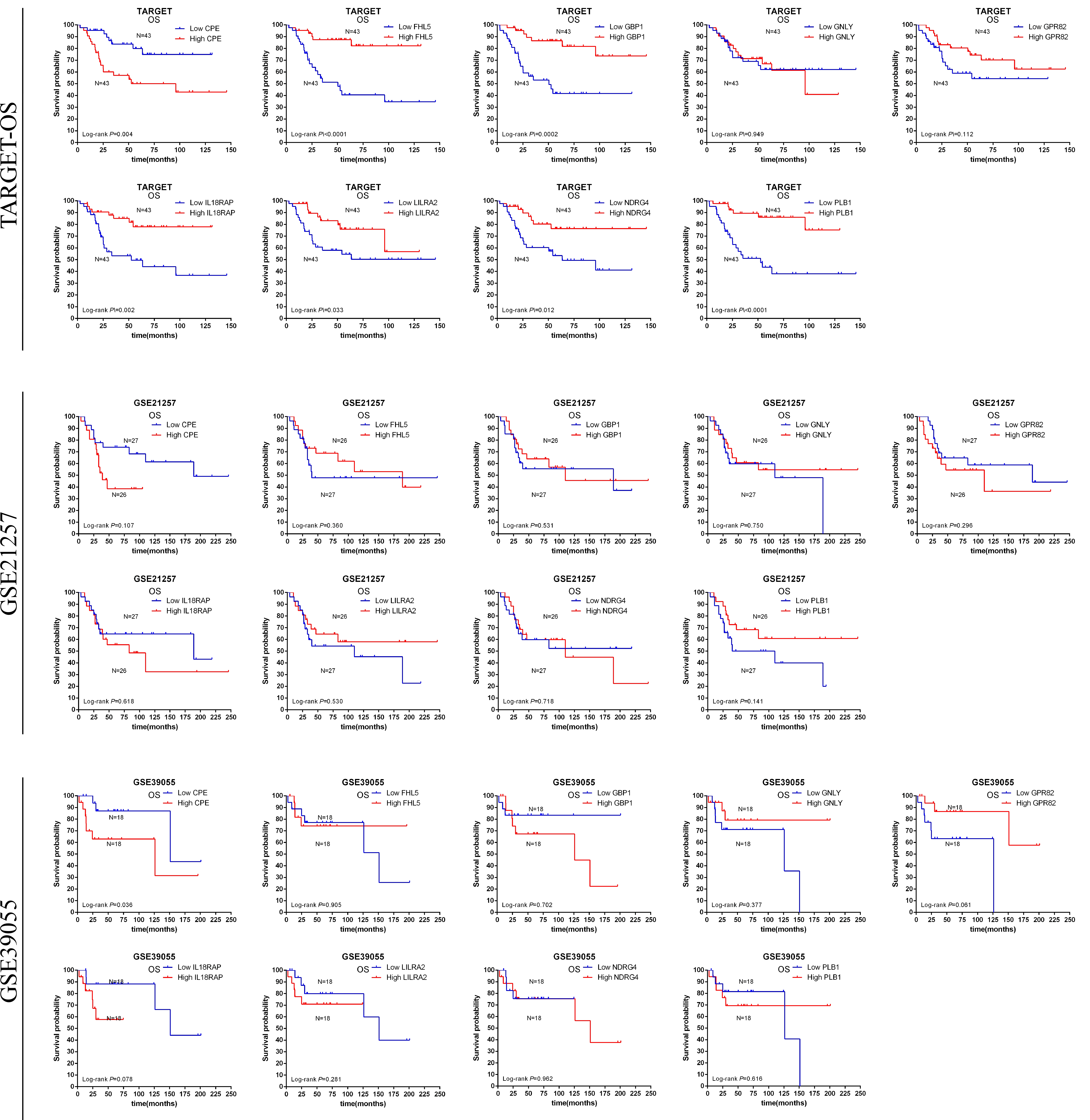


| Supplemental Table 1.Selected M0 macrophage associated genes |
| --- |
| Gene symbol |
| MARCH1,ACSL5,ADAM28,ADCY7,ADORA3,AIF1,AKNA,ALOX5,ALOX5AP,AMICA1,ANKRD22,AOAH,AP1B1,APOBEC3D,APOBEC3G,APOL1,APOL3,APOL6,ARHGAP25,ARHGDIB,ATP8B4,BIN2,BLNK,BST2,BTK,C12orf35,C1orf162,C1QA,C1QB,C1QC,C2,C3,C3AR1,C5AR1,C9orf47,CALML4,CARD11,CASP1,CCDC69,CCL13,CCL18,CCL2,CCL5,CCL8,CCR2,CD14,CD163,CD2,CD27,CD37,CD38,CD3E,CD3G,CD48,CD5,CD53,CD74,CD86,CD8A,CD96,CECR1,CFD,CIDEB,CIITA,CKB,CLEC12A,CLEC7A,CORO7,CPE,CR1,CREG1,CSF3R,CSTA,CTSS,CTSW,CX3CR1,CXCL11,CXCL16,CYBB,DEF6,DENND1C,DENND2D,DENND3,DHCR24,DISC1,DOCK2,DOK2,DOK3,EBI3,EOMES,EPB41L3,F13A1,FAIM3,FCER1G,FCGBP,FCGR2A,FCGR2B,FGD2,FGD4,FGL2,FHL5,FMNL1,FNBP1,FOLR2,FPR1,FUCA1,FXYD6,FYB,GAB3,GBP1,GBP4,GBP5,GCH1,GCNT1,GIMAP4,GIMAP6,GIMAP7,GIMAP8,GNLY,GPR141,GPR34,GPR82,GPR84,GYPC,HAVCR2,HCK,HCLS1,HCST,HESX1,HLA-DMA,HLA-DMB,HLA-DOA,HLA-DRA,HLA-E,HLA-F,HMHA1,HTR2B,ICOS,IFI35,IFI44L,IGSF6,IKBKE,IL18RAP,IL2RA,IQGAP2,IRF1,IRF5,IRF8,ITGAL,ITGAM,ITGB2,JDP2,KCNJ5,KLRB1,KYNU,LAIR1,LAX1,LCK,LCP2,LGMN,LILRA2,LILRB1,LPXN,LRRC25,LRRC33,LRRC8D,LST1,LY86,LY9,MAF,MAP3K5,MAP3K8,MAP7,MERTK,MFNG,MMP9,MNDA,MS4A4A,MS4A6A,MSR1,MYD88,MYO1F,MYO7A,NADK,NAT9,NCF4,NDRG4,NKG7,NPL,OLR1,OSM,P2RX7,P2RY13,P2RY6,PARP3,PARVG,PDE1B,PHF15,PIK3CD,PIK3R5,PILRA,PLB1,PLCG2,PLEK,PLTP,PRF1,PRKCD,PSD4,PSME2,PTAFR,PTGER4,PTPN6,PTPRO,PYCARD,PYHIN1,RAPGEF1,RCSD1,RENBP,RGS18,RHBDF2,RHOH,RNASE1,RNASE2,RNF166,RPS6KA1,RTP4,SAMD3,SAMSN1,SERPINA1,SERPINB9,SGPP1,SIGLEC1,SIGLEC9,SIRPB2,SLA,SLAMF8,SLC11A1,SLC15A3,SLC2A9,SLC43A2,SLC7A7,SLCO2B1,SORL1,SPINT2,ST6GAL1,STAB1,STAT2,STOM,STX11,TAGAP,TAP1,TARP,TBC1D2,TBC1D8,TCN2,TES,TINAGL1,TLR2,TLR4,TLR7,TLR8,TM6SF1,TMEM51,TNFRSF1B,TNFSF12,TNFSF13B,TNFSF8,TRAF3IP3,TRAT1,TREM2,TRIM22,UBE2L6,UCP2,VAV1,VNN2,VSIG4,WARS,WAS |
